# Crystal Structure of Geranylgeranyl Pyrophosphate Synthase (CrtE) Involved in Cyanobacterial Terpenoid Biosynthesis

**DOI:** 10.3389/fpls.2020.00589

**Published:** 2020-05-25

**Authors:** Yuchi Feng, R. Marc L. Morgan, Paul D. Fraser, Klaus Hellgardt, Peter J. Nixon

**Affiliations:** ^1^Department of Chemical Engineering, Imperial College London, London, United Kingdom; ^2^Sir Ernst Chain Building-Wolfson Laboratories, Department of Life Sciences, Imperial College London, London, United Kingdom; ^3^School of Biological Sciences, Royal Holloway, University of London, Egham, United Kingdom

**Keywords:** isoprenoid, prenyltransferase, site-directed mutagenesis, FARM, phylogenetic analysis

## Abstract

Cyanobacteria are photosynthetic prokaryotes that perform oxygenic photosynthesis. Due to their ability to use the photon energy of sunlight to fix carbon dioxide into biomass, cyanobacteria are promising hosts for the sustainable production of terpenoids, also known as isoprenoids, a diverse class of natural products with potential as advanced biofuels and high-value chemicals. However, the cyanobacterial enzymes involved in the biosynthesis of the terpene precursors needed to make more complicated terpenoids are poorly characterized. Here we show that the predicted type II prenyltransferase CrtE encoded by the model cyanobacterium *Synechococcus* sp. PCC 7002 is homodimeric and able to synthesize C20-geranylgeranyl pyrophosphate (GGPP) from C5-isopentenyl pyrophosphate (IPP) and C5-dimethylallyl pyrophosphate (DMAPP). The crystal structure of CrtE solved to a resolution of 2.7 Å revealed a strong structural similarity to the large subunit of the heterodimeric geranylgeranyl pyrophosphate synthase 1 from *Arabidopsis thaliana* with each subunit containing 14 helices. Using mutagenesis, we confirmed that the fourth and fifth amino acids (Met-87 and Ser-88) before the first conserved aspartate-rich motif (FARM) play important roles in controlling chain elongation. While the WT enzyme specifically produced GGPP, variants M87F and S88Y could only generate C15-farnesyl pyrophosphate (FPP), indicating that residues with large side chains obstruct product elongation. In contrast, replacement of M87 with the smaller Ala residue allowed the formation of the longer C25-geranylfarnesyl pyrophosphate (GFPP) product. Overall, our results provide new structural and functional information on the cyanobacterial CrtE enzyme that could lead to the development of improved cyanobacterial platforms for terpenoid production.

## Introduction

Cyanobacteria are a group of gram-negative photosynthetic prokaryotes that perform oxygenic photosynthesis using sunlight to drive the conversion of CO_2_ into a variety of carbon-based compounds. Cyanobacteria are therefore widely considered as promising hosts for the sustainable production of biofuels and commodity and high-value chemicals. Terpenoids are a major class of secondary metabolites with over 70,000 compounds identified (Vickers et al., 2014) including carotenoids, sterols, steroids, saponins, and hormones, which are widely used in a range of applications, including pharmaceuticals, pesticides, fragrances, flavors, and advanced biofuels (Rabinovitch-Deere et al., 2013).

**FIGURE 1:**
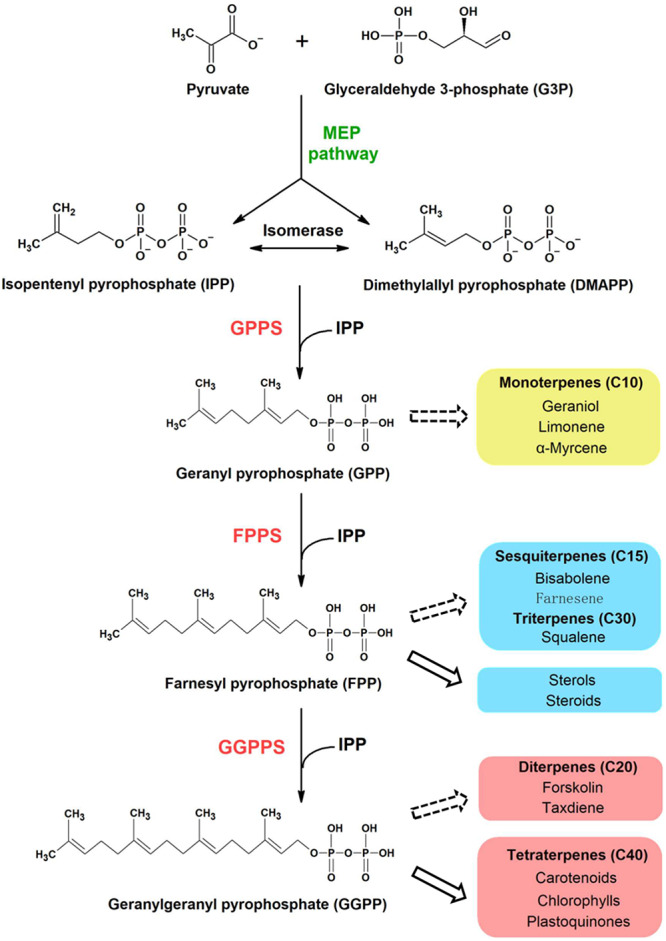
Proposed terpenoid biosynthesis in cyanobacteria via the MEP pathway. Syn7002 CrtE is a hypothesized functional GGPPS, which could use IPP and DMAPP as substrates to generate GGPP as the final product. The arrows with solid lines indicate that the products are generated naturally in cyanobacteria whilst the arrows with dashed lines indicate that the products could be potentially produced from the corresponding substrates through metabolic engineering. MEP, methylerythritol-4-phosphate; GPPS, geranyl pyrophosphate synthase; FPPS, farnesyl pyrophosphate synthase; GGPPS, geranylgeranyl pyrophosphate synthase.

All terpenoids are synthesized from two isomeric C5 building blocks: isopentenyl pyrophosphate (IPP) and dimethylallyl pyrophosphate (DMAPP), both produced via the 2-C-methyl-D-erythritol-4-phosphate (MEP) pathway in cyanobacteria (Figure 1). Prenyltransferase enzymes catalyze the initial condensation reaction between IPP and DMAPP to give C10-geranyl pyrophosphate (GPP), plus the subsequent addition of IPP molecules to give first C15-farnesyl pyrophosphate (FPP), and then C20-geranylgeranyl pyrophosphate (GGPP), which are the precursors of monoterpenoids, sesquiterpenoids and diterpenoids, respectively (Han et al., 2015). A diverse range of polyprenyl pyrophosphate products (>C25) can be further synthesized through addition of different numbers of IPP to the FPP allylic substrate (Wallrapp et al., 2013).

Prenyltransferases are classified as cis (Z) or trans (E) depending on the stereochemistry of the carbon-carbon double bonds in the product (Shimizu et al., 1998). Amino acid sequence alignments of different E- and Z-type enzymes suggests that trans-prenyltransferases have evolved from a common ancestor, whereas cis-prenyltransferases may have a different origin (Chen et al., 1994). In general, trans-prenyltransferases generate products up to C50, which can be further classified into short-chain (C10–C25), medium-chain (C30–C35), and long-chain (C40–C50) (Ogura and Koyama, 1998). GPP, FPP, and GGPP are synthesized by geranyl pyrophosphate synthase (GPPS), farnesyl pyrophosphate synthase (FPPS), and geranylgeranyl pyrophosphate synthase (GGPPS), respectively. C25 compounds, formed by geranylfarnesyl pyrophosphate synthase (GFPPS), are used in the formation of thermophilic archaeal ether-linked lipids, while hexaprenyl pyrophosphate synthase (HexPPS), heptaprenyl pyrophosphate synthase (HepPPS), octaprenyl pyrophosphate synthase (OPPS), solanesyl pyrophosphate synthase (SPPS), and decaprenyl pyrophosphate synthase (DPPS) generate the side chains needed for the formation of quinones (Wang and Ohnuma, 1999).

All these trans-prenyltransferases contain two highly conserved aspartate-rich motifs: the “first aspartate-rich motif” (FARM, DDX_2__–__4_D, where X encodes any amino acid) and the “second aspartate-rich motif” (SARM, DDXXD). In previous studies, a sequential ionization-condensation-elimination catalytic mechanism was proposed, in which FARM and SARM coordinate Mg^2+^ ions to promote elimination of the pyrophosphate group from the allylic substrate (such as DMAPP, GPP, and FPP) to generate a carbocation intermediate, which is subsequently attacked by IPP, bound to positively charged residues in the cavity, to form an adduct that is then deprotonated to form a carbon-carbon double bond (Liang, 2009; Chang et al., 2012).

Among the prenyltransferase enzymes, GGPPS has been considered a bottle-neck enzyme that regulates terpenoid biosynthesis (Jiang et al., 2012; Bai et al., 2016). In the case of photosynthetic organisms, most photosynthesis-related metabolites such as carotenoids and the side chains of chlorophylls and plastoquinones are all derived from GGPP, synthesized by GGPPS (Ruiz-Sola et al., 2016). GGPPS enzymes can be divided into three types: Type-I enzymes contain a large aromatic amino acid at the fourth or fifth position before the FARM; Type-II contain an insertion of two residues in the FARM region but are devoid of aromatic residues at the fourth or fifth position before the FARM; and Type-III are also devoid of aromatic residues just before the FARM but have no extra residues inserted in the FARM (Hemmi et al., 2003; Bouvier et al., 2005; Chang et al., 2006). Structurally, GGPPS enzymes are found mainly as homodimers such as in the archaeon *Geoglobus acetivorans*, the bacterium *Bacteroides thetaiotaomicron*, and the eukaryotes *Plasmodium vivax*, *Saccharomyces cerevisiae*, *Sinapis alba*, and *Arabidopsis thaliana* (Chang et al., 2006; Kloer et al., 2006; Artz et al., 2011; Wallrapp et al., 2013; Wang et al., 2016; Petrova et al., 2018). However, some GGPPS are heterodimers, such as in the cases of *Oryza sativa* and *Mentha piperita* (Chang et al., 2010; Zhou et al., 2017) and higher order oligomers have been described for *Homo sapiens* (Kavanagh et al., 2006).

Little is known about the GGPPS enzymes in photosynthetic microorganisms. In cyanobacteria, sequence comparisons have identified a potential GGPPS (Liang et al., 2006), denoted CrtE because of its role in carotenoid biosynthesis (Yen and Marrs, 1976). An early study identified a gene encoding GGPPS in the thermophilic cyanobacterium *Thermosynechococcus elongatus* (Ohto et al., 1999). However, there is still no information on the structure of this enzyme and how product chain length is regulated.

In this study, we report the first crystal structure of GGPPS (CrtE) from a cyanobacterium – in our case the model strain *Synechococcus* sp. PCC 7002 (Syn7002). Based on the structure, site-directed mutagenesis experiments were carried out to investigate the role of specific amino acids in determining the chain length of the product.

## Materials and Methods

### Cloning, Expression, and Purification

The *crtE* gene (SYNPCC7002_A1085) was amplified by PCR using genomic DNA of Syn7002 and primers CrtE_F (5′- CTCCGCGGTGGTATGGTAGTTGCAGA-3′) and CrtE_R (5′-CGGAATTCTTAGTTTTTACGGTTAACGATG-3′) (restriction sites are underlined). The PCR products were digested with EcoRI and SacII, and the DNA fragments were cloned into the pHUE expression vector (Catanzariti et al., 2004) to give pHUE_CrtE (Supplementary Dataset 1). Protein expression was performed using *E. coli* BL21(DE3) (New England Biolabs, Ltd). Plasmid Usp2-cc which encodes histidine-tagged deubiquitinase was also transformed into *E. coli* BL21(DE3). The His-tagged recombinant proteins were overexpressed in *E. coli* BL21(DE3) in 3 L of Luria-Bertani medium containing 100 mg L^–1^ ampicillin by growing to an optical density at 600 nm of 0.6 with 250 rpm at 37°C (Eppendorf Innova 43 incubator shaker) and then inducing with 0.4 mM isopropyl-1-thio-β-D-galactopyranoside (IPTG). After additional overnight incubation at 18°C, the cells were harvested and disrupted with a T5 cell disruptor (Constant system, GA, United States) using a pressure of 25 kpsi at 5°C in 40 mL of KPN buffer containing 40 mM KH_2_PO_4_/K_2_HPO_4_ (pH 8.0) and 100 mM NaCl. The homogenate was centrifuged with a Beckman Coulter Optima L-100XP ultracentrifuge and a Ti45 rotor (Beckman Coulter, Inc., United States) at 172,000 x *g* for 30 min at 4°C, and the supernatant was recovered as a crude extract. To the 40 mL of supernatant, 2 mL of a 50% slurry of nickel-nitrilotriacetic acid (Ni-NTA) agarose in KPN buffer was added, then placed on a rotary wheel at 4°C for 1.5 h. The lysate/Ni-NTA mixture was centrifuged at 2,000 rpm (805 x *g*) for 2 min (Eppendorf^TM^ 5810 R Centrifuges with a A-4-62 Model rotor), and the Ni-NTA agarose pellet was washed twice with 20 mL of buffer A (25 mM Tris–HCl pH 7.5, 500 mM NaCl) and then twice with 20 mL of buffer A containing 60 mM imidazole. The His-tagged proteins (either deubiquitinase or CrtE fusion protein) were eluted from the Ni-NTA resin in 20 mL of buffer A containing 500 mM imidazole. The purified proteins were dialyzed against 5 L of buffer A at 4°C overnight, and the protein concentration was measured by the Bradford assay using bovine serum albumin (BSA) to generate the standard curve (Zor and Selinger, 1996). Cleavage of the CrtE fusion protein was achieved by incubating with deubiquitinase at a 1:10 molar ratio (enzyme to substrate) overnight at 16°C in 40 mL of buffer A (Catanzariti et al., 2004). Cleaved products were concentrated with a Sartorius 30 K concentrator (Satorius Stedim Lab Ltd, United Kingdom) at 4,000 rpm (3,220 x *g*) at 4°C (Eppendorf^TM^ 5810 R Centrifuge with a A-4-62 Model rotor) until the final volume reduced to about 1 mL and then purified using an ÄKTA Prime FPLC system (GE Healthcare, United Kingdom) equipped with a HiLoad^®^ 16/60 Superdex^®^200 prep grade (pg) (120 mL) column (GE Healthcare, United Kingdom) and equilibrated with 25 mM Tris–HCl (pH 7.5) and 500 mM NaCl buffer in a cold room (4°C). Chromatography was performed at a flow rate of 1 mL min^–1^ and 2 mL fractions were collected. Absorbance of protein was measured at 280 nm (A280). Fractions contained purified CrtE and His-tagged ubiquitin protein were collected and analyzed by SDS-PAGE and immunoblotting (Michoux et al., 2014). Fractions containing CrtE were concentrated with a Sartorius 30 K MWCO concentrator (Sartorius Stedim Lab Ltd, United Kingdom) at 4,000 rpm, 4°C (Eppendorf^TM^ 5810 R Centrifuges with a A-4-62 Model rotor) until the final concentration reached 11.3 mg mL^–1^. The final predicted protein sequence after cleavage is shown in Supplementary Dataset 1.

### Crystallization and Data Collection

Crystallization trials were performed using the sitting drop vapor diffusion method at 16°C. Crystallization drops were dispensed using a Mosquito nanoliter high-throughput robot (TTP Labtech Ltd, United Kingdom) and consisted of 200 nL protein solution and 100 nL reservoir solution. Crystal formation was first observed in the condition containing 0.2 M ammonium acetate, 0.1 M sodium acetate (pH 4.6) and 30% (w/v) PEG 4000 in 2 to 5 days (with an approximate size of 60 μm × 40 μm). After 7 days, crystals were observed in another condition containing 0.1 M sodium citrate (pH 5.5) and 20% (w/v) PEG 3000 (with an approximate size of 500 μm × 100 μm). Crystals were flash-cooled in liquid nitrogen after cryoprotection with 30% (w/v) ethylene glycol.

X-ray diffraction data from native crystals were collected at beamline I03 or I04-1 of the Diamond Light Source (DLS), United Kingdom. The data were processed and scaled using *SCALA* (Evans, 2006) within the xia2-dials package (Winter et al., 2013). In the structural refinements, 5% randomly selected reflections were set aside for calculating R_free_ as a quality monitor (Brünger, 1993).

### Structure Determination and Refinement

The structure of CrtE was solved by molecular replacement (MR) using the structure of GGPPS1 large subunit from *Arabidopsis thaliana* (PDB: 5E8L) (Wang et al., 2016) as a starting model (56.4% amino acid sequence identity). MR was performed with *Phaser* (McCoy et al., 2007). Additional model building was done manually with cycles of WinCoot (Emsley et al., 2010) (Emsley and Cowtan, 2004) and refined in REFMARC5 with using global non-crystallographic symmetry (NCS) restraints (Murshudov et al., 2011). The statistics of the model refinement are summarized in Table 1. Protein molecular graphics were generated with PyMOL and electrostatic surface potential map was calculated using the APBS Electrostatics Plugin (DeLano, 2002). In the structure of Syn7002 CrtE with 2 copies in the asymmetric unit (PDB: 6SXL), we identified the following residues in chain A: 8 to 172, 180 to 241 and 263 to 300 of the predicted 302 residues; chain F: 8 to 241 and 253 to 300. In the structure of Syn7002 CrtE with 6 copies in the asymmetric unit, the identified residues of each monomer were similar, covering the range from 9 to 232 and 254 to 297.

**TABLE 1:** Data collection and refinement statistics for the Syn7002 CrtE crystal structures (in brackets are the statistics for the high-resolution shells).

	**Syn7002CrtE (six copies, PDB: 6SXN)**	**Syn7002CrtE (two copies, PDB: 6SXL)**
Diffraction source	I04-1, DLS	I03, DLS
Wavelength (Å)	0.9159	0.9763
Temperature (K)	100	100
Detector	PILATUS 6M	PILATUS 6M
Space group	P2_1_2_1_2_1_	P2_1_2_1_2_1_
a, b, c (Å)	102.56, 122.97, 134.19	70.20, 89.47, 107.32
α, β, γ (°)	90.00, 90.00, 90.00	90.00, 90.00, 90.00
Resolution range (Å)	68.02–2.66	68.82–2.50
R_merge_	0.06 (0.75)	0.03 (0.45)
R_meas_	0.08 (1.1)	0.04 (0.63)
R_pim_	0.06 (0.75)	0.03 (0.44)
CC1/2	0.99 (0.48)	0.99 (0.77)
Average I/σ(I)	11.76 (1.63)	11.72 (1.94)
Completeness (%)	99.24 (95.35)	99.97 (100.00)
Multiplicity	2.0 (2.0)	2.0 (2.0)
Total no. of reflections	98338 (9428)	48009 (4718)
No. of unique reflections	49329 (4674)	24024 (2358)
Refinement		
R_work_/R_free_ (%)	22.8/28.5	24.3/30.6
Root-mean-square deviations		
Bond lengths (Å)	0.008	0.009
Bond angles (°)	1.556	1.840
Ramachandran plot (%)		
Most favored regions (%)	96.00	93.47
Allowed regions (%)	3.08	6.33
Outliers (%)	0.92	0.19
Average B-factor	73.73	77.69
Protein	73.76	77.74
Ligands	n/a	114.88
Solvent	57.95	57.92

### *In vitro* CrtE Assays

CrtE prenyltransferase activity was determined *in vitro* as described previously (Jones et al., 2013). The incubation mixture contained, in a total volume of 200 μL, 50 mM Tris–HCl (pH 7.5), 5 mM MgCl_2_, 1 mM DTT, 46 μM [1-^14^C] IPP (1.85 GBq mmol^–1^), 0.5% (v/v) Triton X-100 and purified protein, and 10 μM DMAPP. Samples were incubated for 1 h at 30°C and reactions stopped by cooling on ice and the addition of 200 μL of 1 M NaCl. Samples were extracted in 2 mL of water-saturated butanol overnight at -20°C. Butanol extractable products were dephosphorylated by adding potato acid phosphatase (1 unit) (Sigma-Aldrich, United Kingdom) to 1 mL of extract and incubating in 0.2% (v/v) Triton X-100 and 4 mL of methanol in a total volume of 10 mL at 37°C overnight on a shaking platform water bath. Samples were extracted in 10 mL of 10:90 (v/v) diethyl ether/petroleum ether (boiling point 40–60°C) and the organic phase dried under nitrogen. Reaction products (geraniol, farnesol, and geranylgeraniol) were identified by thin-layer chromatography (TLC) using reversed-phase C18 F254S plates (Merck, United Kingdom), with a mobile phase of 19:1 (v/v) acetone/water. The radio-labeled products were identified by autoradiography, and alcohol standards (geraniol, farnesol, geranylgeraniol, and solanesol) (Sigma-Aldrich, United Kingdom) were visualized by exposure to iodine vapor.

### Site-Directed Mutagenesis of GGPPS

Reverse PCR was used to linearize the original pHUE_CrtE plasmid with the mutagenic oligonucleotides (shown below), and the re-circulation of the plasmids was achieved by using In-fusion HD Cloning Kit (Thermo Fisher Scientific, United Kingdom). All mutations were confirmed by DNA sequencing (GENWIZ, United Kingdom). The correct constructs were subsequently transformed into *E. coli* BL21 (DE3) for protein expression. The primers designed to produce the desired point mutations were: M87Y, 5′-GAG ATGATCCACACC**TAT**TCTTTAATCCATGATGATCTGCC-3′ and 5′- GGTGTGGATCATCTCCAGG-3′; S88F, 5′-ATGATC CACACCATG**TTT**TTAATCCATGATGATCTGCCCG-3′ and 5′- CATGGTGTGGATCATCTCCA-3′; M87Y/S88F, 5′- GAG ATGATCCACACC**TATTTT**TTAATCCATGATGATCTGCCCG -3′ and 5′- GGTGTGGATCATCTCCAGG-3′; A96Y, 5′-CATGA TGATCTGCCC**TAT**ATGGACAATGACGATCTCCGT-3′ and 5′- GGGCAGATCATCATGGATTAAAG-3′, V161M, 5′-GGGG CCGAAGGCCTC**ATG**GGTGGCCAAGTGGTGGAT-3′ and 5′- GAGGCCTTCGGCCCCC-3′; A96Y/V161M, 5′-CATGATGAT CTGCCC**TAT**ATGGACAATGACGATCTCCGT-3′, 5′- GAG GCCTTCGGCCCCC -3′, 5′-GGGGCCGAAGGCCTC**ATG**G GTGGCCAAGTGGTGGAT-3′ and 5′- GGGCAGATCATCA TGGATTAAAG-3′; M87A, 5′-GAGATGATCCACACC**GCC** TCTTTAATCCATGATGATCTGCC-3′ and 5′- GGTGTGGAT CATCTCCAGG-3′; K53A, 5′-CTCCTGGCTGGGGGA**GCC**CG GCTACGCCCGATTCTTT-3′ and 5′- TCCCCCAGCCAGG AGG-3′. These substituted codons are frequently used in Syn7002. The mutated positions are indicated in boldface type.

### Bioinformatic Techniques

Full-length amino-acid sequences of several prenyltransferases (for detailed sequence information, see Supplementary Dataset 2) were obtained from the UniProt database^1^ and aligned with Aliview software using the MUSCLE program^2^ and displayed with ESPript^3^. GGPPS (CrtE) protein sequences used for phylogenetic analysis and analysis of amino-acid residue conservation were downloaded from UniProt (see Supplementary Dataset 3). The phylogenetic tree was constructed using the Neighbor-joining method with the PhyML 3.0^4^. The default parameters of the above software were used. The tree was visualized using Interactive Tree of Life^5^. Analysis of the conservation of GGPPS from cyanobacteria, algae and plants was performed using the ConSurf server^6^.

## Results

### Comparison of CrtE to Other Prenyltransferases

**FIGURE 2:**
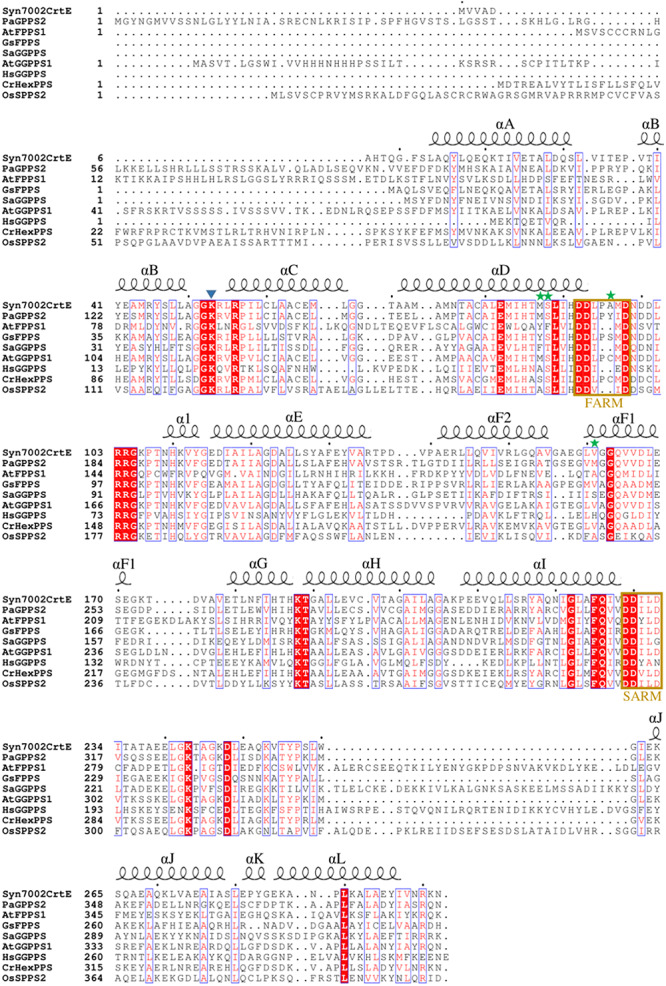
Alignment of amino acid sequences of Syn7002, *Synechococcus* sp. PCC 7002 CrtE; Pa, *Picea abies* GPPS; At, *Arabidopsis thaliana* FPPS1 and GGPPS1; Gs, *Geobacillus stearothermophilus* FPPS; Sa, *Sulfolobus acidocaldarius* GGPPS; Hs, *Homo sapiens* GGPPS; Cr, *Capsella rubella* HexPPS; and Os, *Oryza sativa* SPPS2. Strictly conserved residues are highlighted by using red background and conservatively substituted residues are boxed. The secondary structure α-helices of Syn7002 CrtE are shown above the aligned sequences. The blue triangle indicates the Lys residue predicted to bind to IPP; the green stars indicate the amino acid residues mutated in this study.

For Syn7002, three hypothesized prenyltransferases genes have been annotated in the genome sequence in UniProt: *crtE* (*SYNPCC7002*_A1085), *sdsA* (*SYNPCC7002_*A0580), and *uppS* (*SYNPCC7002_*A0099), which are considered to function as GGPPS, SPPS and undecaprenyl pyrophosphate synthase (UPPS) enzymes, respectively. In Figure 2, the amino acid sequence of CrtE from Syn7002 is compared to GPPS, FPPS, GGPPS, HexPPS, and SPPS enzymes from various organisms. CrtE, in common with other trans-prenyltransferases, contains the two highly conserved FARM and SARM regions (Tarshis et al., 1996; Narita et al., 1999) and contains a two amino-acid insertion (Pro-95 and Ala-96) in the FARM characteristic of a Type-II GGPPS (Hemmi et al., 2003).

### Expression, Purification, and Crystallization of Syn7002 CrtE

To produce CrtE in *E. coli* for structural studies, the CrtE protein was expressed as an N-terminal histidine-tagged ubiquitin fusion protein using the high-level expression vector pHUE (Catanzariti et al., 2004). Recombinant CrtE protein was over-expressed in *E. coli* and purified first using Ni-NTA agarose (Supplementary Figure 1) and after cleavage of the His-tagged ubiquitin-CrtE fusion by deubiquitinase (Supplementary Figure 2), the liberated CrtE protein was then purified by size-exclusion chromatography and analyzed by SDS-PAGE (Supplementary Figures 3A,C). The molecular mass of CrtE was estimated to be about 61 kDa based on the elution volume (Supplementary Figure 3B), close to the predicted mass of 65 kDa for a dimer.

We obtained CrtE crystals in two different crystallization conditions (Supplementary Figures 3D,E). The crystal formed in 0.2 M ammonium acetate, 0.1 M sodium acetate (pH 4.6) and 30% (w/v) PEG 4000 had 6 copies in the asymmetric unit, and the crystal generated in 0.1 M sodium citrate (pH 5.5) and 20% (w/v) PEG 3000 contained 2 copies in the asymmetric unit. In both cases CrtE crystals had the same space group - P2_1_2_1_2_1_. Among the structurally characterized prenyltransferase enzymes, the predicted CrtE from Syn7002 shows 56.3% sequence identity with the large subunit of *A. thaliana* GGPPS1 (PDB: 5E8L). This was successfully used as a starting model to determine the structure of CrtE by molecular replacement. In the present study, we determined the structures of Syn7002 CrtE with 6 copies in the asymmetric unit in its apo form to a resolution of 2.66 Å (PDB: 6SXN) and with 2 copies in the asymmetric unit in its apo form to a resolution of 2.50 Å (PDB: 6SXL) (Table 1). Structure-based sequence alignments between the CrtE homodimer in PDB:6SXN (chain C and E) and PDB:6SXL (chain A and B) showed a root mean square difference (RMSD) of 0.471 Å over 520 residues (2531 atoms superposed) performed using PyMOL (Supplementary Figure 4). The three CrtE dimers (CE, BD, and AF) in PDB: 6SXN have corresponding RMSD of 0.256 Å (CE and BD, 2791 atoms superposed), 0.278 Å (CE and AF, 2827 atoms superposed) and 0.413 Å (AF and BD, 2953 atoms superposed), respectively. The RMSD between the CrtE monomer in PDB: 6SXN (chain C) and PDB:6SXL (chain F) is 0.420 Å over 258 residues (1825 atoms superposed).

### Overall Structure of Syn7002 CrtE

**FIGURE 3:**
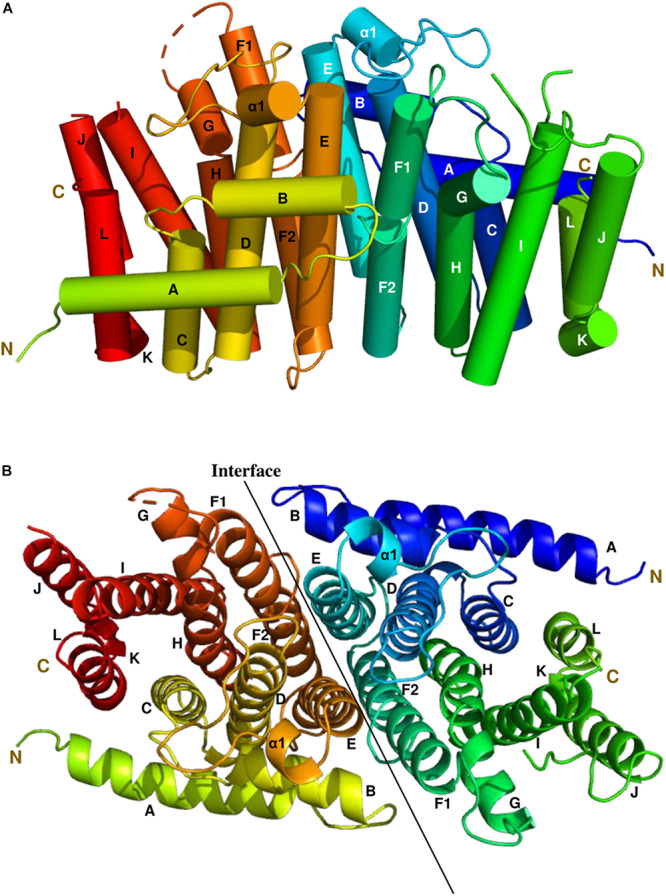
The side and top views model of the Syn7002 CrtE homodimer is shown using a cylinder **(A)** and ribbon diagram **(B)** (PDB: 6SXN, chain C and E), respectively. N, N-terminal; C, C-terminal. Helices are labelled A to L.

The most complete structure was obtained with PDB: 6SXN chain C, in which all 302 residues apart from the first eight N-terminal residues, twenty-two residues from 233 to 254 and the last five C-terminal residues could be identified in the electron density map. Each CrtE monomer is composed of 14 α-helices (A to L, and α1) joined by connecting loops (Figure 3). Helix F is broken in the middle to give helices F1 and F2, respectively. A short helix α1 (Asn-109-Tyr-113) was observed between helix D and E. A similar structure has been reported for *M. piperita* GPPS and *Sinapis alba* GGPPS, and is thought to be involved in the protein conformational changes needed for substrate binding and product release (Kloer et al., 2006; Chang et al., 2010). The two aspartate-rich motifs, FARM and SARM, are located in helix D and H, respectively, forming a large catalytic cavity for allyl substrate binding (Tarshis et al., 1996; Guo et al., 2004). α-helices (D, E, F1, and F2) form a large tunnel-shaped pocket which is hypothesized to be involved in prenyl chain elongation according to previous structural studies (Wallrapp et al., 2013; Wang et al., 2016). The inner surface of the pocket is filled with hydrophobic amino acids (Ala-80, Ile-84, Met-87, Leu-126, Phe-130, Leu-151, Val-155, and Leu-160) which, based on previous studies on prenyltransferase enzymes, are predicted to determine the chain length of the final product (Tarshis et al., 1996). In addition, residues in helices E, F1 and F2 at the homodimer interface are non-polar. Notably, the side chain of Phe-130 in helix E is involved in a π-π interaction with its counterpart in the other subunit (Supplementary Figure 5), which also has been found in *A. thaliana* GFPPS and *Sulfolobus solfataricus* HexPPS (Sun et al., 2005; Wang et al., 2016).

**FIGURE 4:**
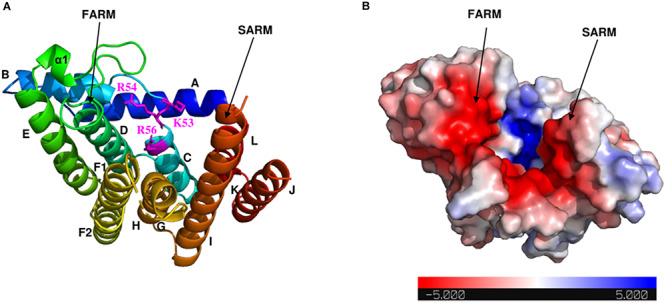
Electrostatic surface potential map of Syn7002 CrtE monomer (PDB: 6SXN, chain C). **(A)** Ribbon diagram of the Syn7002 CrtE monomer with positively charged Lys and Arg side chains highlighted. **(B)** The electrostatic surface potential map of the Syn7002 CrtE monomer. The color is coded from red to blue according to charge potential from –5 to 5 kT/e. The potential was calculated using the APBS plugin in PyMOL. FARM, first conserved aspartate-rich motif; SARM, second conserved aspartate-rich motif.

As shown in the electrostatic surface potential map (Figure 4), there are a number of positively charged amino acid residues (Lys-53, Arg-54, and Arg-56) located at the bottom of the catalytic cavity, which are proposed to bind the IPP substrate (Wang et al., 2016). The negatively charged FARM and SARM regions sit atop of the cavity flanking the positively charged bottom. Binding sites for Mg^2+^ and DMAPP could not be obtained in this study, but have been reported in previous structural studies of GGPPS from *S. cerevisiae*, *Plasmodium falciparum*, and *Corynebacterium glutamicum* (Guo et al., 2007; No et al., 2012; Wallrapp et al., 2013).

### Regulation of Product Chain Length

**FIGURE 5:**
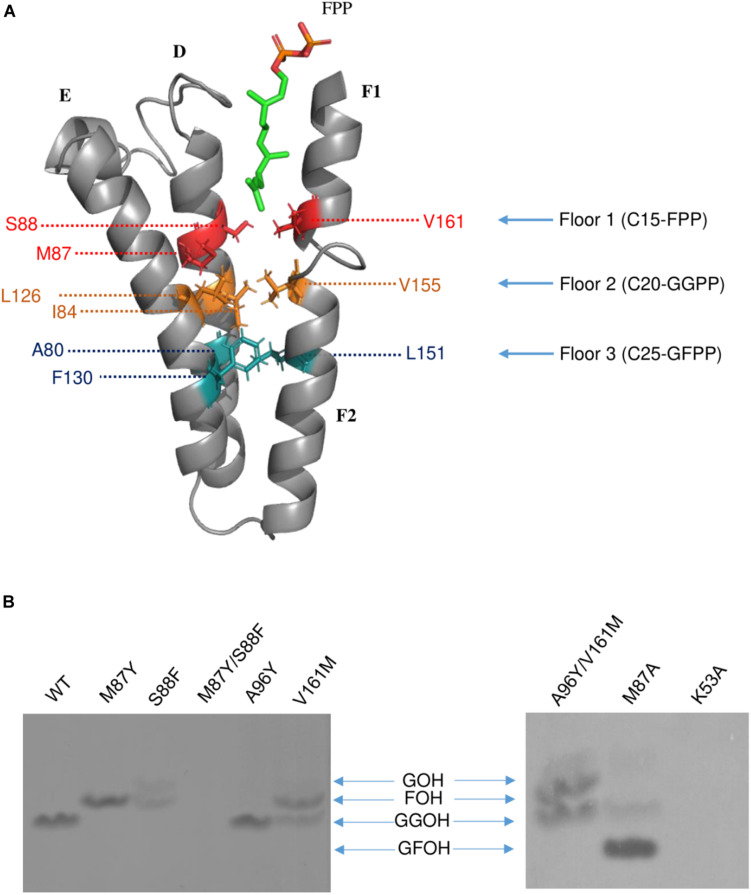
**(A)** The hypothesized “Three-floors” model based on the Syn7002 GGPPS structure built with PyMOL. Residues on floor 1 (red), floor 2 (yellow) and floor 3 (cyan). FPP is used as an example to elucidate the mechanism of product chain-length elongation. **(B)** *In vitro* assay of wild type and mutated Syn7002 CrtE protein. DMAPP and ^14^C-IPP were used as allylic substrates in all reactions, and the products were analyzed by TLC. GOH, geraniol; FOH, farnesol; GGOH, geranylgeraniol; GFOH, geranylfarnesol.

Previous studies have demonstrated that the side chains of residues in the hydrophobic pocket (formed by helices D, E, F1, and F2) could form a “floor” that blocks product elongation and so determine the product chain length (Sun et al., 2005; Han et al., 2015). Based on the structure of wild type Syn7002 CrtE, an equivalent “floor” model was built, and amino-acid residues predicted to determine product chain length were identified (Figure 5A). Site-directed mutagenesis of CrtE was performed to test the first “floor” of this model; therefore, Met-87 and Ser-88 on helix D, and Val-161 on helix F1, were replaced by residues with larger (Phe and Tyr) or smaller (Ala) side chains.

As pure CrtE WT and CrtE WT fusion gave the same result in the TLC chromatography profile (Supplementary Figure 6), the variant His-tagged CrtE fusion proteins were directly used for their enzyme assays after purification using immobilized Ni-affinity chromatography and dialysis. Enzyme assays were conducted with purified wild-type and variant fusion proteins (Supplementary Figure 7) using [1-^14^C] IPP and DMAPP as substrates as described in materials and methods. The radio-labeled products were dephosphorylated, and the generated alcohols were observed on TLC (Figure 5B). Under the same reaction conditions, the final product of wild type CrtE was C20-GGPP. Variants M87Y and S88F both shifted the final product from GGPP to C15-FPP, but S88F produced less FPP and some C10-GPP compared to the products of M87Y. However, no product was detected in the mutated enzyme (M87Y/S88F) in which both residues were changed, possibly because the enzyme kinetics were dramatically affected, or the final product was volatile. When Met-87 was mutated to Ala, somewhat surprisingly, the longer geranylfarnesyl pyrophosphate molecule (GFPP, C25) was generated as a major product, with traces of GGPP. The second residue before the conserved GQ motif (Val-161) on helix D, which is also hypothesized to be involved in the first floor formation (Hemmi et al., 2003), was mutated to Met, and found to produce similar amounts of FPP and GGPP.

Type-II Syn7002 CrtE has two extra residues (Pro-95 and Ala-96) inserted within the FARM region. These two residues are located in the loop between helices D and E, and spatially on the top of the elongation pocket in the structure. According to the sequence alignment in Figure 1, Pro-95 in the FARM is highly conserved in Type-II prenyltransferases, but the second additional residue shows more variability. For instance, PaGPPS has a bulky Tyr residue at the second position, whereas the other Type-II prenyltransferases (Syn7002 CrtE, GsFPPS, AtGGPPS, and CrHexPPS) have a smaller residue such as Cys, Ser and Ala. To determine whether a bulky residue at this position could interrupt the elongation reaction, to give rise to GPP, we mutated CrtE Ala-96 to Tyr, but GGPP was still formed. These data indicate that the size of the second extra amino acid in the FARM does not critically affect product chain-length elongation. We also tried to mimic PaGPPS by making a version of CrtE with A96Y/V161M, so that the mutated enzyme has the same first “floor” and the same two residue insertion as that found in PaGPPS. But like the V161M single variant, similar amounts of FPP and GGPP were produced and there was no evidence for formation of GPP. Mutation of the predicted IPP binding site (Lys-53) was achieved by replacing it with Ala but no products were detected.

### Phylogenetic Relationship of Photosynthetic Organisms GGPPSs

**FIGURE 6:**
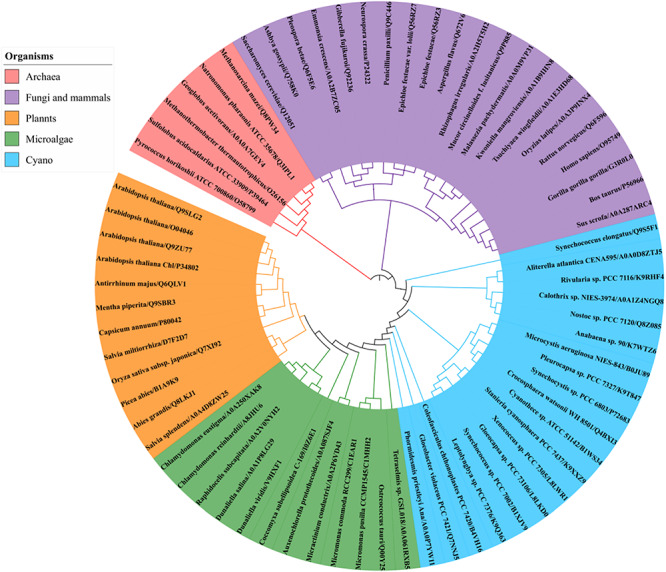
Phylogenetic analysis of GGPPS from archaea, cyanobacteria, algae, plants, fungi, and mammals using the Neighbor-joining method of PhyML 3.0 (http://www.atgc-montpellier.fr/phyml/) based on their amino acid sequences. Alphanumeric codes to the right of the organisms’ names correspond to accession numbers on the UniProt database.

A BLAST search of the UniProt online protein database of archaea, cyanobacteria, algae, higher plants, fungi and mammal sequences (Supplementary Dataset 3) using Syn7002 CrtE (GGPPS) was conducted to study the evolutionary relationships. A phylogenetic analysis (Figure 6), based on the degree of sequence similarity, revealed that all the GGPPSs have evolved from a common ancestor and form three distinct clades (Type-I GGPPS in archaebacteria, Type-II GGPPS in photosynthetic organisms, and Type-III GGPPS found in fungi and mammals). GGPPS from photosynthetic organisms (Type-II) and GGPPS from fungi and mammals (Type-III) belong to different clades but may share a common ancestor.

**FIGURE 7:**
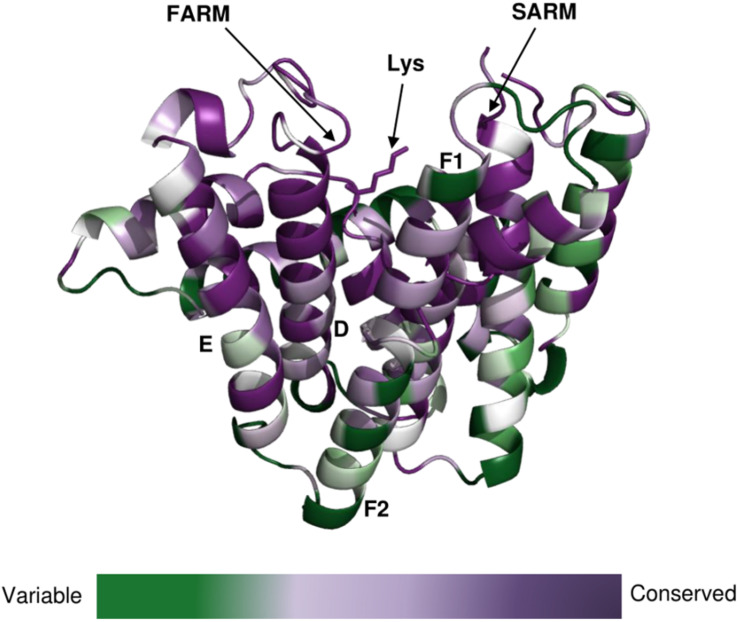
Conservation of amino-acid residues in GGPPS in representative cyanobacteria, algae and higher plants mapped onto the structure of CrtE from Syn7002 CrtE (6SXN, chain C) performed with the ConSurf server (https://consurf.tau.ac.il/). Residue conservation is colored in a gradient spectrum in such a way that the least conserved region is colored in green, and the most conserved region is colored in purple. A conserved Lys (Lys-53), proposed to bind the substrate IPP, is indicated.

The clade of photosynthetic organisms was divided into three clusters (cyanobacteria, algae, and plants). Analysis of the sequence conservation of Type-II GGPPSs was conducted based on sequence alignments and the determined Syn7002 GGPPS structure (PDB: 6SXN) using the ConSurf server (Figure 7). The essential functional domains such as short helix α1, FARM, SARM, the ligand-binding region and the residues in the product elongation tunnel are highly conserved, which indicates that GGPPSs in photosynthetic organisms have a very close evolutionary relationship and are likely to display similar mechanisms for catalysis and for controlling product chain length.

## Discussion

In this study, we have demonstrated that the CrtE enzyme encoded by the model cyanobacterium Syn7002 codes for a Type-II GGPPS. A phylogenetic analysis indicates that Type-II GGPPS is widely found in photosynthetic organisms. Cyanobacteria and algae encode one GGPPS (CrtE) and higher plants usually have multiple GGPPS enzymes, for example *A. thaliana* has 10 GGPPSs (Ruiz-Sola et al., 2016). Uniquely, the thermophilic cyanobacterium *T. elongatus* possesses a FPPS in addition to a GGPPS (Ohto et al., 1999). Syn7002 GGPPS is a homodimer and its three-dimensional structure is most similar to the large subunit of heterodimeric *A. thaliana* GGPPS1. Heterodimeric and heterotetrameric GGPPSs are found in higher plants, with the large subunit involved in catalysis and the small subunit in product release (Chang et al., 2010; Wang et al., 2016).

Syn7002 GGPPS contains the two highly conserved aspartate-rich motifs (FARM and SARM), which are important for substrate binding and catalysis in prenyltransferases (Song and Poulter, 1994; Koyama et al., 1996). In most trans-prenyltransferase structures, dimer interactions have been observed between helices E and F (F1 and F2) (Guo et al., 2004; Noike et al., 2008; Han et al., 2015; Wang et al., 2016). Van der Waals interactions and stacking (π-π) interactions between the hydrophobic residues on these two helices contribute to dimerization (Sun et al., 2005). Previous studies have demonstrated that a positively charged residue Lys at the bottom of the catalytic cavity is responsible for IPP binding: Lys-36 in *S. cerevisiae* GGPPS (PDB: 2E8U), Lys-60 in *Plasmodium vivax* GGPPS (3LDW), and Lys-47 in *Trypanosoma brucei* FPPS (PDB: 3DYF) (Guo et al., 2007; Zhang et al., 2009; Artz et al., 2011). In addition, Koyama et al. (1996) replaced Lys-47 with Ile in *B. stearothermophilus* FPPS and found the affinity for substrate IPP was decreased. In Syn7002 GGPPS, replacement of Lys-53 by Ala led to no detectable formation of product. Arg-56, which is highly conserved in prenyltransferases, is also ideally placed to interact with IPP, as also observed for *S. cerevisiae* GGPPS (Guo et al., 2007).

A “three-floor” model has been proposed to explain how the length of the final product is controlled in prenyltransferases (Wang et al., 2016). The size of residues at each floor determines the length of the final product. For example, “Floor one” determines if the final product is C15-FPP, “Floor two” if it is C20-GGPP and “Floor three” if it is C25-GFPP (Figure 5A). From these models and corresponding site-directed mutagenesis experiments, it is generally believed that the size of the residues located four and five positions before the FARM determines the chain length of the product, with a large side chain forming a “floor” that blocks further product elongation (Ohnuma et al., 1996; Narita et al., 1999; Guo et al., 2004; Sun et al., 2005; Szkopi and Danuta, 2005; Ling et al., 2007; Han et al., 2015). According to our structural models, Syn7002 CrtE has two small residues and one medium sized Met residue on the first floor, and its second floor is formed from Ile-84, Leu-126 and Val-155 (Figure 5A). Surprisingly, replacement of Met-87 on the first floor with Ala generated a longer product C25-GFPP, suggesting that Met-87 has a role in forming the first floor but its side chain also indirectly impacts the second floor. An identical result was also observed previously for the equivalent Met to Ala mutation in AtGGPPS1 (Wang et al., 2016) and, indeed, the predicted first floor residues are exactly the same in Syn7002 CrtE and AtGGPPS1 and the second floor residues are very similar with Ile, Leu, and Val found in Syn7002 CrtE and Ile, Leu, and Ile in AtGGPPS1.

The residues before another conserved motif G(Q/E) on helix F1 are also considered to regulate chain termination (Hemmi et al., 2003; Kawasaki et al., 2003). Our sequence alignment (Figure 2) demonstrated that the size of the second residue before the G(Q/E) motif in the short-chain prenyltransferases (like PaGPPS and HsGGPPS) is larger than that in the medium/long-chain prenyltransferases. In addition, our Syn7002 GGPPS structure showed that the second residue (Val-161) before the conserved GQ motif is more likely to generate a “floor” with Met-87 and Ser-88 according to its spatial position. Mutating Val-161 to Met partially blocked the reaction and generated more FPP and less GGPP compared to the products of wild type GGPPS. A similar mutagenesis study was performed with *S. acidocaldarius* HexPPS: a point mutation at this position (S140H, S140F, and S140Y) resulted in the shortening of the final products (Hemmi et al., 2003). Collectively, these results indicate that the second residue before the conserved G(Q/E) motif is also involved in determining product chain length. The third amino acid (L151) before the G(Q/E) motif of *A. thaliana* GGPPS1 has also been reported to be important for “floor” formation, with mutation of this residue (L151F) leading to the formation of shorter products (GPP and FPP) and less GGPP (Wang et al., 2016).

Cyanobacteria have been considered as green microbial cell factories for producing high value-added products. However, the yield of short chain terpenoids is limited by the small pool sizes of their precursors GPP and FPP (Kiyota et al., 2014), as the major product of CrtE is GGPP. We have shown here that mutation of CrtE can alter product specificity. In principle, it is possible to use this knowledge to make cyanobacterial strains with larger pools of FPP or GFPP which could be useful platforms for the production of sesquiterpenoids, triterpenoids and sesterterpenoids.

## Data Availability Statement

Two protein structures were elucidated and submitted to the RCSB Protein Data Bank: the structures of Syn7002 CrtE with six copies in the asymmetric unit in its apo form (PDB accession: 6SXN) and with two copies in the asymmetric unit in its apo form (PDB accession: 6SXL).

## Author Contributions

YF performed the experimental work. RM collected the X-ray diffraction data and solved the structures. PF contributed reagents, materials, and analysis tools for TLC analysis. YF, RM, PF, and PN designed the experiments. All authors listed have contributed to data interpretation, preparation, and writing of the manuscript.

## Conflict of Interest

The authors declare that the research was conducted in the absence of any commercial or financial relationships that could be construed as a potential conflict of interest.

## Footnotes

1 https://www.uniprot.org/

2 https://www.ebi.ac.uk/Tools/msa/muscle/

3 http://espript.ibcp.fr/ESPript/ESPript/

4 http://www.atgc-montpellier.fr/phyml/

5 https://itol.embl.de/

6 https://consurf.tau.ac.il/
