# Supplementary material for: Crystal Structure of Geranylgeranyl Pyrophosphate Synthase (CrtE) Involved in Cyanobacterial Terpenoid Biosynthesis
Source: Front Plant Sci. 2020 May 25;11:589. doi: 10.3389/fpls.2020.00589 (PMC7261888; doi:10.3389/fpls.2020.00589)
Supplement: Supplementary file 1 [file Data_Sheet_1.docx]

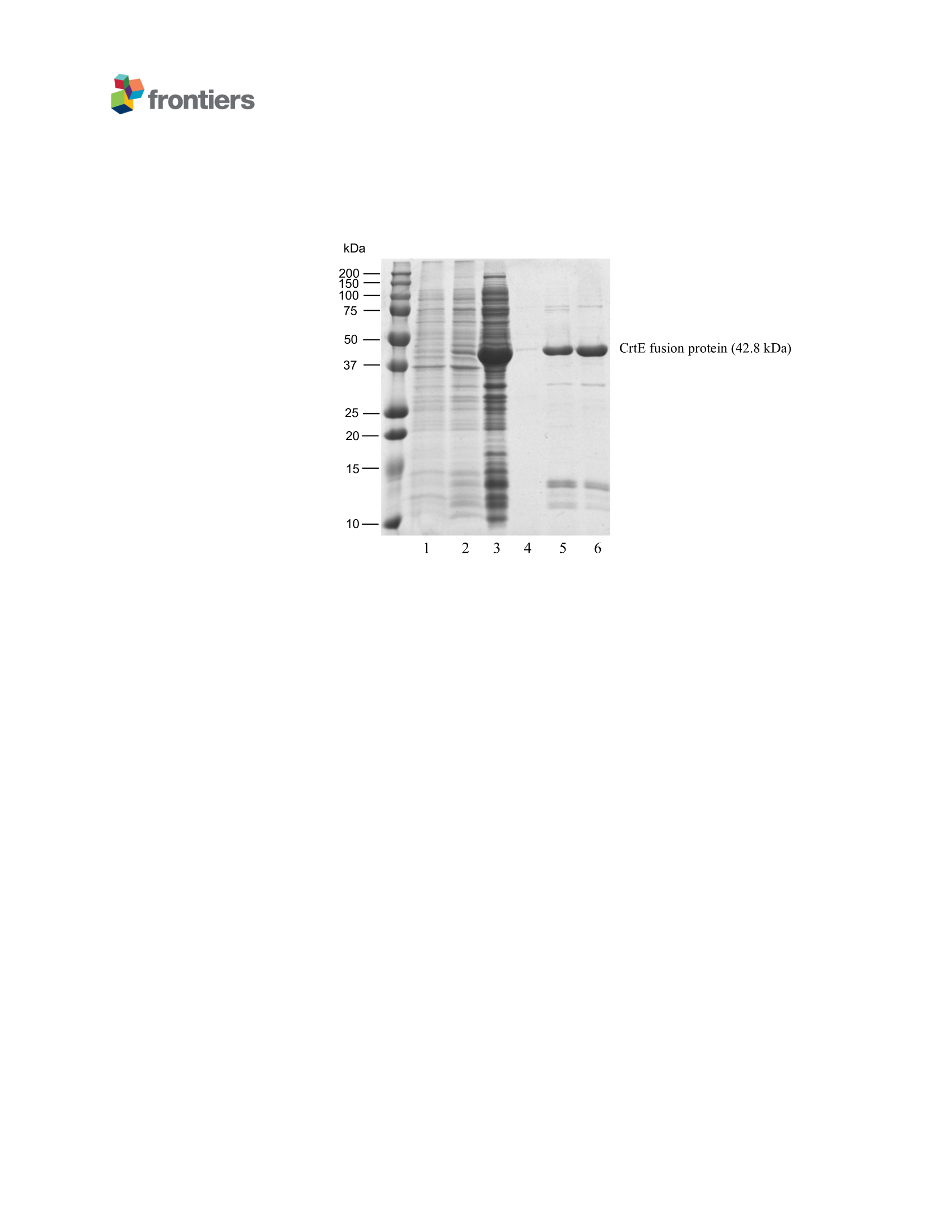
Supplementary Material

**Supplementary Figure 1.** Expression and purification of Syn7002 CrtE fusion protein. Lane 1: pre-induction; 2: post-induction; 3: all soluble fractions by cell disruptor; 4: fraction from wash buffer without imidazole; 5: fraction from wash buffer with 60 mM imidazole; 6: Fraction from elution buffer with 500 mM imidazole. Fractions were resolved by 15% SDS-PAGE and Coomassie blue staining. Ladder: Bio-rad Prestained Dual Color Protein Standard.


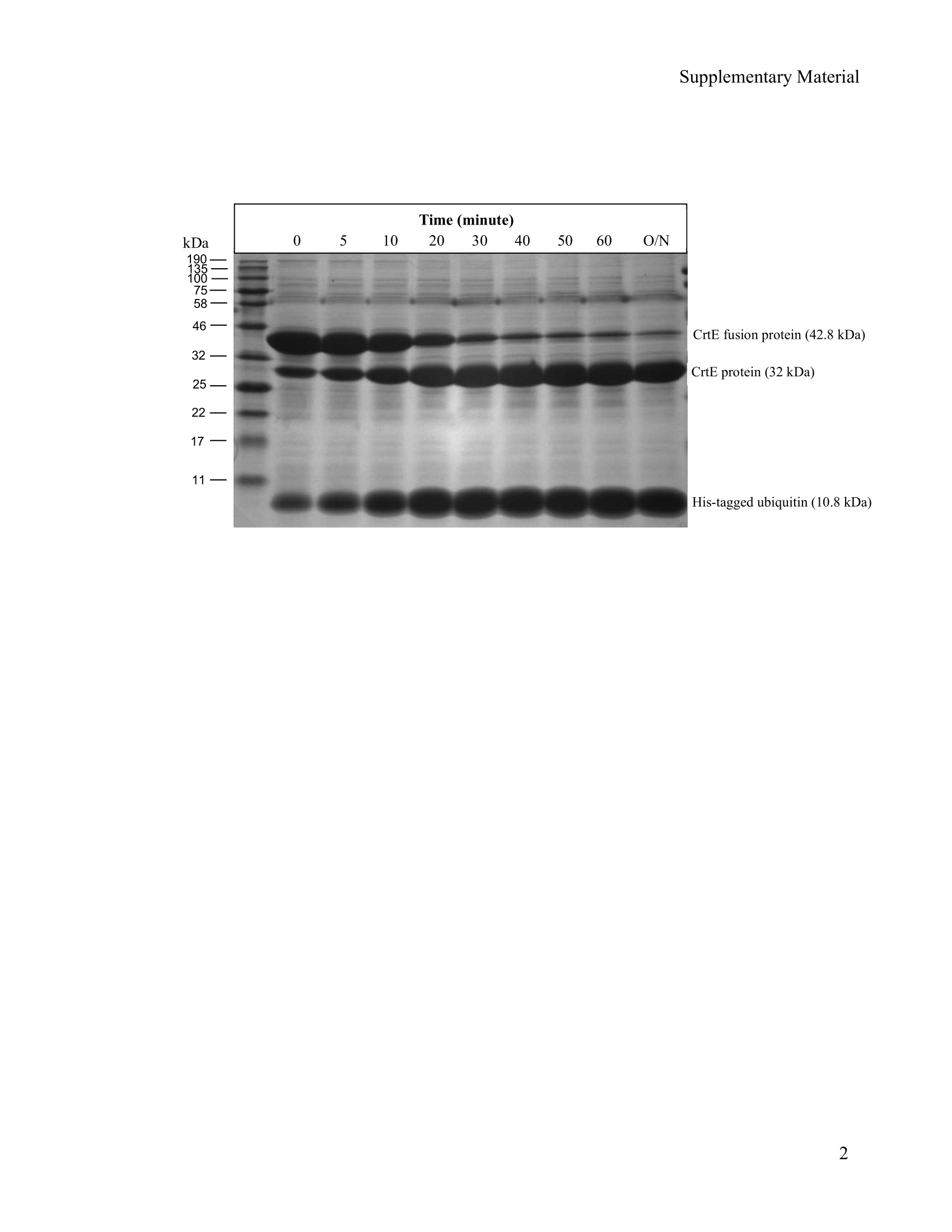


**Supplementary Figure 2.** Digestion of Syn7002 CrtE fusion protein with deubiquitinase over a 60-min time course at a 1: 100 molar ratio. Samples were taken at 0, 5, 10, 20, 30, 40, 50, 60 min and overnight (O/N) (shown above lanes), then resolved by 15% SDS-PAGE and Coomassie blue staining. The position of fusion proteins and the two cleaved products are indicated on the right. Ladder: Blue Prestained Protein Standard (Broad range).


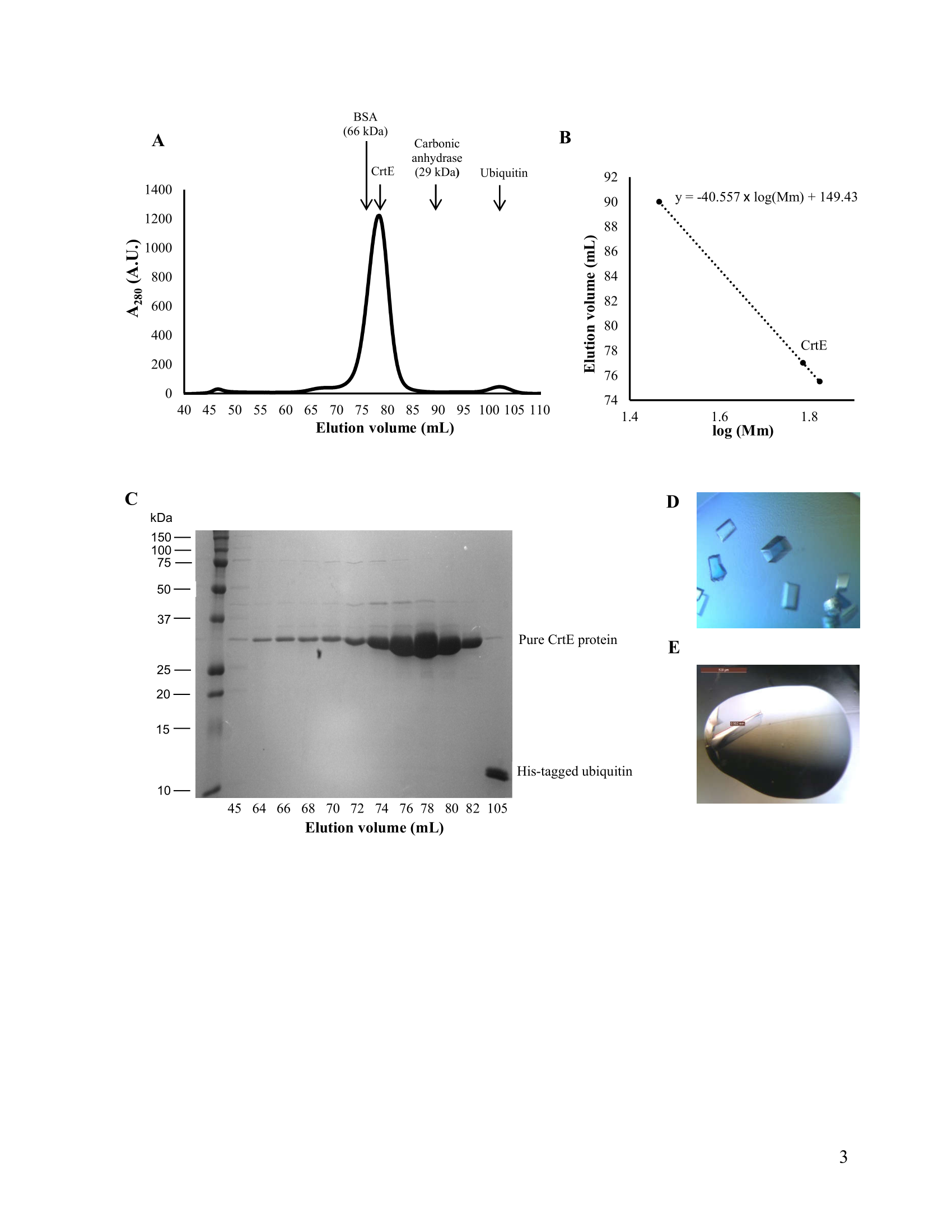


**Supplementary Figure 3.** Purification of cleaved products by size exclusion chromatography (A), BSA retention volume: 75 mL, carbonic anhydrase retention volume: 90 mL. Gel filtration protein standard curve used to estimate the molecular weight of the native CrtE. BSA (66.5 kDa) and carbonic anhydrase from bovine erythrocytes (29 kDa) were used. The elution volume (mL) was recorded as a function of log molecular mass (Mm). The data were plotted on a semi-log plot and fit with a linear function (B). The estimated molecular mass of native CrtE is about 61.1 kDa, close to the predicted mass of 65 kDa for a dimer. Collected fractions were analysed by 15% SDS-PAGE and Coomassie blue staining (C). Images of crystals formed in different conditions. 0.2 M Ammonium acetate, 0.1 M Sodium acetate pH 4.6, 30 % (w/v) PEG 4000 (D); 0.1 M Sodium citrate pH 5.5, 20 % (w/v) PEG 3000 (E).


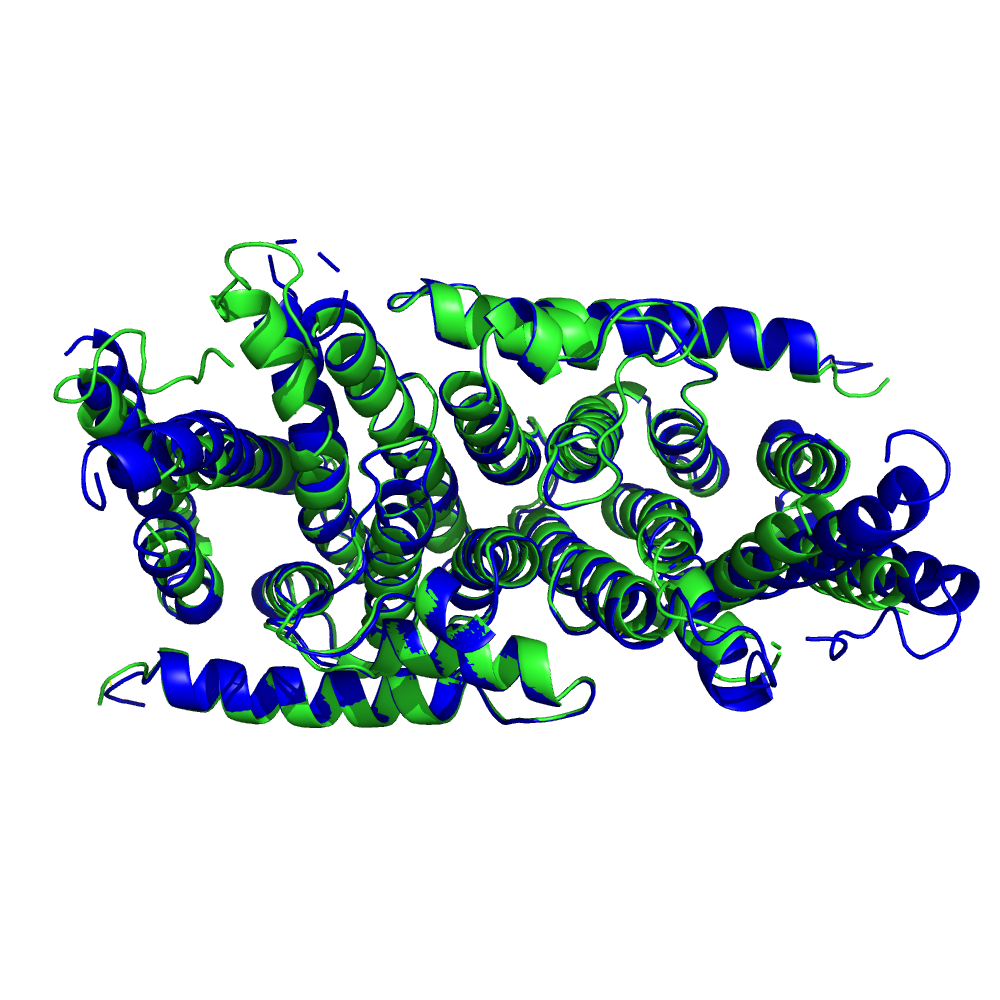


**C**

**N**

**N**

**C**

**Supplementary Figure 4.** Syn7002 CrtE apo Structures’ alignment between CrtE apo form with 6 copies in the asymmetric unit (green, PDB: 6SXN, chain C and E) and 2 copies in the asymmetric unit (blue, PDB: 6SXL, chain A and F). N, N-terminal; C, C-terminal. These figures were produced using PyMOL.


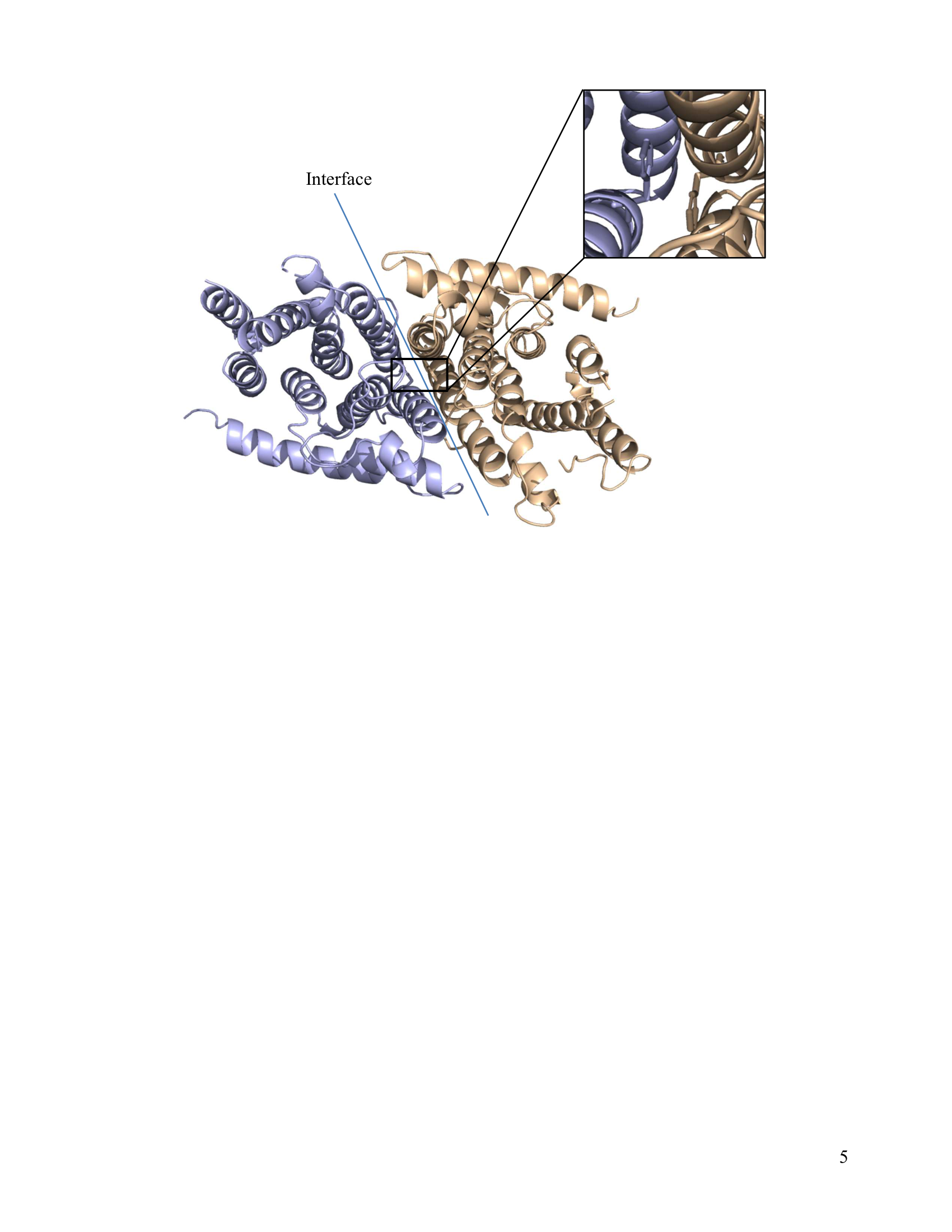


**Supplementary Figure 5.** π-π interactions of residues Phe-130 from Syn7002 CrtE two monomers (PDB: 6SXN, chain C and E)


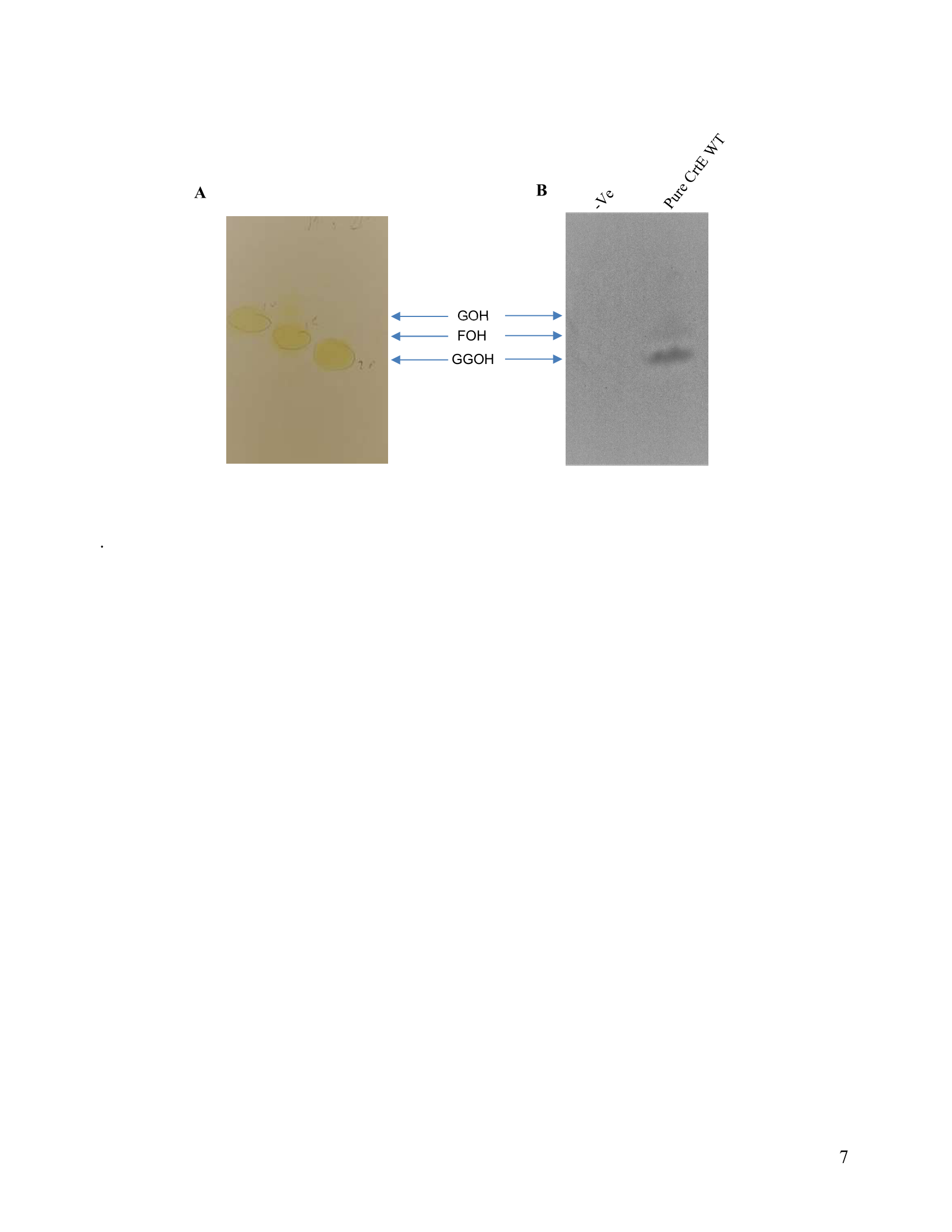


**Supplementary Figure 6.** *In vitro* assay of pure CrtE wild type protein. Standards (A) and reaction products (B) were analysed by TLC. Standards GOH: geraniol; FOH: farnesol; GGOH: geranylgeraniol. The standards were visualized by exposure to iodine vapor

.

**
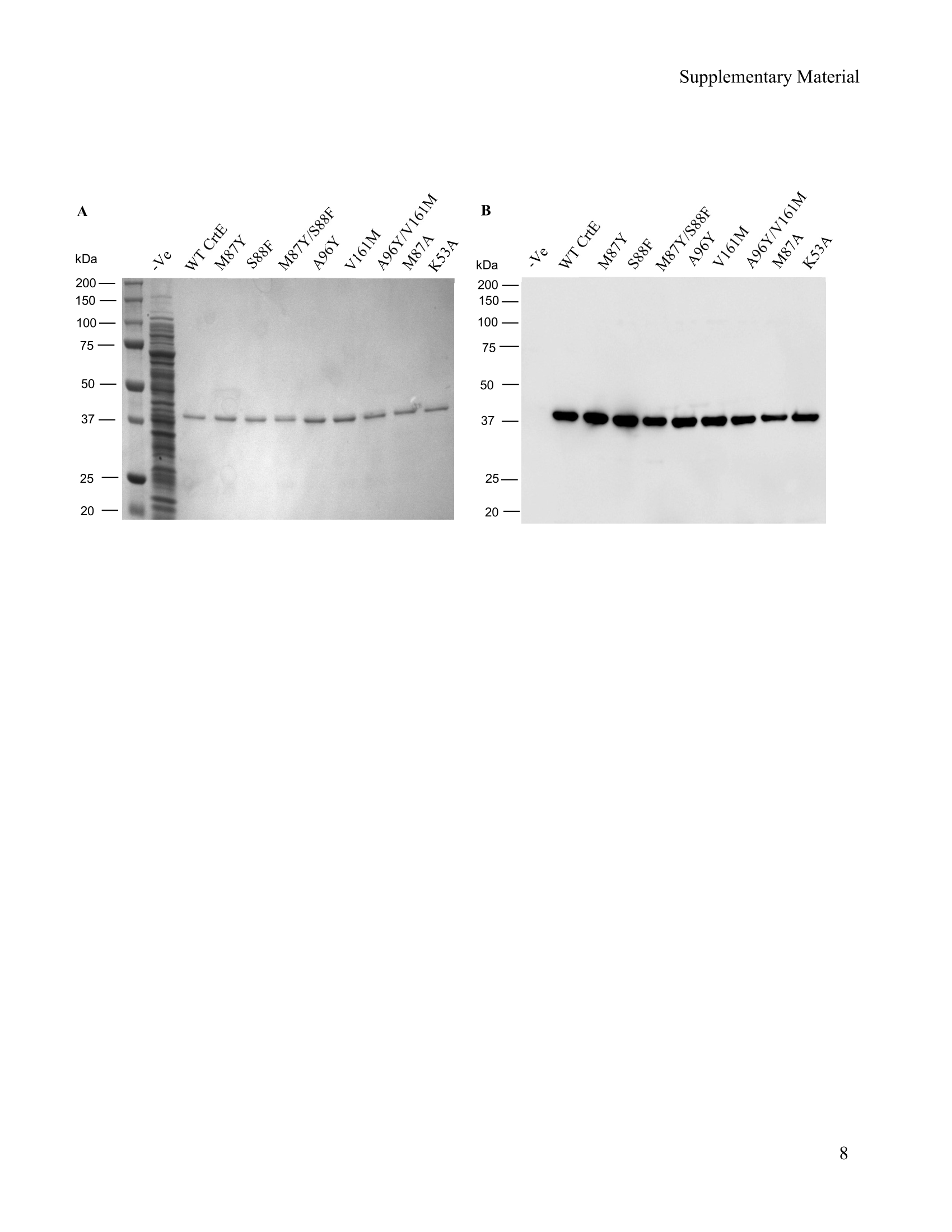
**

**Supplementary Figure 7.** Expression and purification of Syn7002 CrtE WT and variants. Purified fusion proteins were analysed by SDS-PAGE (A) and immunoblotting (B). –Ve: *E. coli* BL21 (DE3) wild type all soluble proteins. 6x-His Tag monoclonal antibody was used in the Western Blot (Merk, UK).

**Supplementary dataset 1.** The amino acid sequence of pHUE_CrtE protein. Final protein sequence after cleavage is shown in red.

MGSSHHHHHHSSGLVPRGSHMQIFVKTLTGKTITLEVEPSDTIENVKAKIQDKEGIPPDQQRLIFAGKQLEDGRTLSDYNIQKESTLHLVLRLRGGMVVADAHTQGFSLAQYLQEQKTIVETALDQSLVITEPVTIYEAMRYSLLAGGKRLRPILCLAACEMLGGTAAMAMNTACALEMIHTMSLIHDDLPAMDNDDLRRGKPTNHKVYGEDIAILAGDALLSYAFEYVARTPDVPAERLLQVIVRLGQAVGAEGLVGGQVVDLESEGKTDVAVETLNFIHTHKTGALLEVCVTAGAILAGAKPEEVQLLSRYAQNIGLAFQIVDDILDITATAEELGKTAGKDLEAQKVTYPSLWGIEKSQAEAQKLVAEAIASLEPYGEKANPLKALAEYIVNRKN

**Supplementary dataset 2**. The prenyltransferase enzyme sequences used for sequence alignment shown in Figure 2.

> *Synechococcus* sp. PCC 7002 CrtE (B1XJV9)

MVVADAHTQGFSLAQYLQEQKTIVETALDQSLVITEPVTIYEAMRYSLLAGGKRLRPILCLAACEMLGGTAAMAMNTACALEMIHTMSLIHDDLPAMDNDDLRRGKPTNHKVYGEDIAILAGDALLSYAFEYVARTPDVPAERLLQVIVRLGQAVGAEGLVGGQVVDLESEGKTDVAVETLNFIHTHKTGALLEVCVTAGAILAGAKPEEVQLLSRYAQNIGLAFQIVDDILDITATAEELGKTAGKDLEAQKVTYPSLWGIEKSQAEAQKLVAEAIASLEPYGEKANPLKALAEYIVNRKN

> *Picea abies* GPP2 (B1A9K6)

MGYNGMVVSSNLGLYYLNIASRECNLKRISIPSPFHGVSTSLGSSTSKHLGLRGHLKKELLSHRLLLSSTRSSKALVQLADLSEQVKNVVEFDFDKYMHSKAIAVNEALDKVIPPRYPQKIYESMRYSLLAGGKRVRPILCIAACELMGGTEELAMPTACAIEMIHTMSLIHDDLPYIDNDDLRRGKPTNHKVFGEDTAIIAGDALLSLAFEHVAVSTSRTLGTDIILRLLSEIGRATGSEGVMGGQVVDIESEGDPSIDLETLEWVHIHKTAVLLECSVVCGAIMGGASEDDIERARRYARCVGLLFQVVDDILDVSQSSEELGKTAGKDLISDKATYPKLMGLEKAKEFADELLNRGKQELSCFDPTKAAPLFALADYIASRQN

> *Arabidopsis thaliana* FPPS1 (Q09152)

MSVSCCCRNLGKTIKKAIPSHHLHLRSLGGSLYRRRIQSSSMETDLKSTFLNVYSVLKSDLLHDPSFEFTNESRLWVDRMLDYNVRGGKLNRGLSVVDSFKLLKQGNDLTEQEVFLSCALGWCIEWLQAYFLVLDDIMDNSVTRRGQPCWFRVPQVGMVAINDGILLRNHIHRILKKHFRDKPYYVDLVDLFNEVELQTACGQMIDLITTFEGEKDLAKYSLSIHRRIVQYKTAYYSFYLPVACALLMAGENLENHIDVKNVLVDMGIYFQVQDDYLDCFADPETLGKIGTDIEDFKCSWLVVKALERCSEEQTKILYENYGKPDPSNVAKVKDLYKELDLEGVFMEYESKSYEKLTGAIEGHQSKAIQAVLKSFLAKIYKRQK

> *Geobacillus_stearothermophilus* FPPS (Q08291)

MAQLSVEQFLNEQKQAVETALSRYIERLEGPAKLKKAMAYSLEAGGKRIRPLLLLSTVRALGKDPAVGLPVACAIEMIHTYSLIHDDLPSMDNDDLRRGKPTNHKVFGEAMAILAGDGLLTYAFQLITEIDDERIPPSVRLRLIERLAKAAGPEGMVAGQAADMEGEGKTLTLSELEYIHRHKTGKMLQYSVHAGALIGGADARQTRELDEFAAHLGLAFQIRDDILDIEGAEEKIGKPVGSDQSNNKATYPALLSLAGAKEKLAFHIEAAQRHLRNADVDGAALAYICELVAARDH

> *Sulfolobus acidocaldarius* GGPPS (P39464)

MSYFDNYFNEIVNSVNDIIKSYISGDVPKLYEASYHLFTSGGKRLRPLILTISSDLFGGQRERAYYAGAAIEVLHTFTLVHDDIMDQDNIRRGLPTVHVKYGLPLAILAGDLLHAKAFQLLTQALRGLPSETIIKAFDIFTRSIIIISEGQAVDMEFEDRIDIKEQEYLDMISRKTAALFSASSSIGALIAGANDNDVRLMSDFGTNLGIAFQIVDDILGLTADEKELGKPVFSDIREGKKTILVIKTLELCKEDEKKIVLKALGNKSASKEELMSSADIIKKYSLDYAYNLAEKYYKNAIDSLNQVSSKSDIPGKALKYLAEFTIRRRK

>*Arabidopsis thaliana* GGPPS1 (P34802)

MASVTLGSWIVVHHHNHHHPSSILTKSRSRSCPITLTKPISFRSKRTVSSSSSIVSSSVVTKEDNLRQSEPSSFDFMSYIITKAELVNKALDSAVPLREPLKIHEAMRYSLLAGGKRVRPVLCIAACELVGGEESTAMPAACAVEMIHTMSLIHDDLPCMDNDDLRRGKPTNHKVFGEDVAVLAGDALLSFAFEHLASATSSDVVSPVRVVRAVGELAKAIGTEGLVAGQVVDISSEGLDLNDVGLEHLEFIHLHKTAALLEASAVLGAIVGGGSDDEIERLRKFARCIGLLFQVVDDILDVTKSSKELGKTAGKDLIADKLTYPKIMGLEKSREFAEKLNREARDQLLGFDSDKVAPLLALANYIAYRQN

> *Homo sapiens* GGPPS (O95749)

MEKTQETVQRILLEPYKYLLQLPGKQVRTKLSQAFNHWLKVPEDKLQIIIEVTEMLHNASLLIDDIEDNSKLRRGFPVAHSIYGIPSVINSANYVYFLGLEKVLTLDHPDAVKLFTRQLLELHQGQGLDIYWRDNYTCPTEEEYKAMVLQKTGGLFGLAVGLMQLFSDYKEDLKPLLNTLGLFFQIRDDYANLHSKEYSENKSFCEDLTEGKFSFPTIHAIWSRPESTQVQNILRQRTENIDIKKYCVHYLEDVGSFEYTRNTLKELEAKAYKQIDARGGNPELVALVKHLSKMFKEENE

> *Capsella rubella* HexPPS (R0G5U4)

MDTREALVYTLISFLLSFQLVFWRFRPRCTKVMSTLRLTRHVNIRPLNSPKSYKFEFMSYMVNKAKSVNKALEEAVPLREPVLKIHEAMRYTLLSDGKRVRPMLCLAACELVGGHESTAMSVACGMEMLHASSLILDDLPCMDDDCLRRGKPTNHMVFGEGISILASDALIALAVQKAATSTLLDVPPERVLRAVKEMVKAVGTEGLVAGQAADLAGEGMGFDSNTALEHLEFIHIHKTAALLEAAAVTGAIMGGGSDKEIEKLRSYARCIGLMFQVVDDVLDVTKSSEELGKTAGKDLIAGKLTYPRLMGVEKSKEYAERLNREAREHLQGFDSDKVAPLLSLADYVLNRKN

> *Oryza sativa* SPPS2 (Q75HZ9)

MLSVSCPRVYMSRKALDFGQLASCRCRWAGRSGMRVAPRRRMPCVCFVASPSQPGLAAVDVPAEAISSARTTTMIPERISVSSLLEVVSDDLLKLNNNLKSLVGAENPVLVSAAEQIFGAGGKRLRPALVFLVSRATAELAGLLELTTEHQRLAEIIEMIHTASLIHDDVIDDSGMRRGKETIHQLYGTRVAVLAGDFMFAQSSWFLANLENIEVIKLISQVIKDFASGEIKQASTLFDCDVTLDDYLLKSYYKTASLLASSTRSAAIFSGVSTTICEQMYEYGRNLGLSFQVVDDILDFTQSAEQLGKPAGSDLAKGNLTAPVIFALQDEPKLREIIDSEFSESDSLATAIDLVHRSGGIRRAQELAKEKGDLALQNLQCLPKSQFRSTLENVVKYNLQRID

**Supplementary dataset 3.** The GGPPS protein sequences used for the phylogenetic analysis shown in Figure 5 and amino acid residues conservation analysis shown in Figure 6.

>*Malassezia pachydermatis* (A0A0M9VP31)

MDEAQHAWYEDVRAYVDMRGPWPEAHEKAVSEPYRYLDAHPGKELRSQLIDAFNVWIHVPPTQLEYVKCVVRRLHTASLLMDDVEDNSDLRRGVPAAHTIYGVPQTVNTANYVYFQVLSDIVQQQPDAMPAMIDELVRLHRGQGMDLFWRDSLQCPTEQEYVDMVVNKTGGLFRIALRLMLASAPTPPTVDLAPLANVIGIYFQIRDDYVNLQSTQLAQHKGFCEDLTEGKFSFPILHAIRTAEPDRTLLHILRQRTTHIETKKYAIEYMERVTHSFSYTRTVLQALAQQAEDELTHLEHVLGANPALHSILDVLARPC

>*Penicillium paxilli* (Q9C446)

MSYILAEALNFVRRGISHLNYWGASHSLSADNYWESNFQGFPRLLSDSSKAPSTIRTVQVLEDDVDDIAIQYNKIVRGPLDYLLAIPGKDIRSKLIDSFNIWLQLPEEKLSIVKDIINLLHTASLLIDDIQDASRLRRGKPVAHDVYGVAQTINSANYAYYLQQARLKEIGDPRAFEIFTRSLLDLHLGQGMDLYWRDMVVCPTEEEYTRMVMYKTGGLFNLALDLMRIQSRKNTDFSKLVELLGVIFQIRDDYMNLQSGLYAEKKGLMEDLTEGKFSYPIIHSIRASPESSELLDILKQRTEDEAVKIRAVKIMESTGSFQYTRETLSRLSAEARGYVKKLETSLGPNPGIHKILDLLEVEYPTNEKGRV

>*Epichloe festucae var. lolii* (Q56RZ7)

MTMAANDFPFQCQEKKSYSQPSLVYCNGNIAETYLEEKVLTAPLDYLRALPSKDIRSGLTDAINEFLRVPEEKVLVIKRIIDLLHNASLLIDDIQDSSKLRRGVPVAHHIFGIAQTINSANLAYFIAQRELEKLTNPRAFAIYNEELINLHRGQGMELHWRESLHCPTEDEYLRMIQKKTGGLFRLAIRLLQGESASDDDYVSLIDTLGTLFQIRDDYQNLQSDIYSKNKGYCEDLTEGKFSYPVIHSIRSRPGDVRLINILKQRSEDVMVKQYAVQHIESTGSFAFCQNKIQSLVEQAREQLAALENSSSCGGPVRDILDKLAIKPRANIEVE

>*Epichloe festucae* (Q56RZ3)

MTMAANDFPFQCQEKKSYSQPSLVYCNGNIAETYLEEKVLTAPLDYLRALPSKDIRSGLTDAINEFLRVPEEKVLVIKRIIDLLHNASLLIDDIQDSSKLRRGVPVAHHIFGIAQTINSANLAYFIAQRELEKLTNPRAFAIYNEELINLHRGQGMELHWRESLHCPTEDEYLRMIQKKTGGLFRLAIRLLQGESASDDDYVSLIDTLGTLFQIRDDYQNLQSDIYSKNKGYCEDLTEGKFSYPVIHSIRSRPGDVRLINILKQRSEDVMVKQYAVQHIESTGSFAFCQNKIQSLVEQAREQLAALENSSSCGGPVRDILDKLAIKPRANIEVE

>*Aspergillus flavus* (Q672V6)

MISGVPDRWKVVASSLSSNLDASYPTSSSLSTEPIDTRSSSPQGSASTPVDKEKIIRGPVDYLLKCPGKDIRRKLMQAFNEWLRIPEDRLNIIAEIVGLLHTASLLIDDIQDSSKLRRGIPVAHSIFGVAQTINSANYAYFAAQEKLRELNRPKAYEIFTEELLRLHRGQGMDLYWRDSLTCPTEEEYIEMISNKTGGLFRLAIKLMQLESEVTSDFLGLVDLLGVIFQIRDDYQNLQSDLYSKNKGFCEDLTEGKFSFLIIHSINSNPGNQQLLNILRQRSEEESVKKYAVEYIRSTGSFAYCQDRLASFLHEAKMMVNVLEDNVGFSKGIYDILAFLL

>*Pleospora betae* (Q6F5E6)

MAYTVEPREHSKNTTLPTVAMPPSPPSSFSASFGPFRYDTKEVNFDHWTSTKEKVVTGPYDYIAAKPGKEVRTLLLACFDEWLQVPPESLEVIGQVVRMLHTASLLIDDIQDNSELRRGKPVAQNIFGTALTINSANYVYFLALEKLNSLKNPNITDIFTEELLRLHRGQAMDLYWRDTLTCPTEEEYFEMVANMTGGLFWLMYRMMNAESSMPIDLLPVVELLGVIFQVLDDYKNLCSREYGKLKGFGEDLTEGKFSFPVIHSIRSNPEDLQLLHVLQQKSSNEHVKLYAIEIMESTGSLEYTKHVVENIVSQIQEIIYSTDEGQGRGKGILDLLHKITRLS

>*Oryzias latipes* (A0A3P9INX4)

MAGASEAASDRILLEPYKYLLQLPGKQVRTKLAQAFNHWLNIPDDKLQVIIGVTEMLHNASLLIDDIEDNSKLRRGFPVAHSIYGIPSVINSANYVYFLGLEKVLTLDHPEAVRVFTRQLLELHRGQGLDIHWRDTYTCPTEEEYRSMVLQKTGGLFGLAVGLMQLFSEWKQDLKPLLDTMGLFFQIRDDYANLSSSEYSENKSFCEDLTEGKFSFPTIHAIWSRPESTQVQNILRQRTENMDIKRYCVDYLEKVGSFAYTRHTLRDLEVEAYRLIRDLGGNPQLEALVKQLSQIICLKKKKKFSKGEFYGIN

>*Rattus* *norvegicus* (Q6F596)

MEKTKEKAERILLEPYKYLLQLPGKQVRTKLSQAFNHWLKVPEDKLQIIIEVTEMLHNASLLIDDIEDSSKLRRGFPVAHSIYGVPSVINSANYVYFLGLEKVLTLDHPDAVKLFTRQLLELHQGQGLDIYWRDTYTCPTEEEYKAMVLQKTGGLFGLAVGLMQLFSDYKEDLKPLLDTLGLFFQIRDDYANLHSKEYSENKSFCEDLTEGKFSFPTIHAIWSRPESTQVQNILRQRTENIDIKKYCVQYLEDVGSFEYTRYTLRELEAKAYKQIEACGGNPSLVALVKHLSKMFTEENE

>*Homo sapiens* (O95749)

MEKTQETVQRILLEPYKYLLQLPGKQVRTKLSQAFNHWLKVPEDKLQIIIEVTEMLHNASLLIDDIEDNSKLRRGFPVAHSIYGIPSVINSANYVYFLGLEKVLTLDHPDAVKLFTRQLLELHQGQGLDIYWRDNYTCPTEEEYKAMVLQKTGGLFGLAVGLMQLFSDYKEDLKPLLNTLGLFFQIRDDYANLHSKEYSENKSFCEDLTEGKFSFPTIHAIWSRPESTQVQNILRQRTENIDIKKYCVHYLEDVGSFEYTRNTLKELEAKAYKQIDARGGNPELVALVKHLSKMFKEENE

>*Gorilla gorilla gorilla* (G3R0L0)

MEKTQETVQRILLEPYKYLLQLPGKQVRTKLSQAFNHWLKVPEDKLQMIIEVTEMLHNASLLIDDIEDNSKLRRGFPVAHSIYGIPSVINSANYVYFLGLEKVLTLDHPDAVKLFTRQLLELHQGQGLDIYWRDNYTCPTEEEYKAMVLQKTGGLFGLAVGLMQLFSDYKEDLKPLLNTLGLFFQIRDDYANLHSKEYSENKSFCEDLTEGKFSFPTIHAIWSRPESTQVQNILRQRTENIDIKKYCVHYLEDVGSFEYTRNTLKELEAKAYKQIDARGGNPELVALVKHLSKMFKEENE

>*Bos taurus* (P56966)

MEKTQETVQRILLEPYKYLLQLPGKQVRTKLSQAFNHWLKVPEDKLQIIIEVTEMLHNASLLIDDIEDNSKLRRGFPVAHSIYGIPSVINSANYVYFLGLEKVLTLNHPDAVKLFTRQLLELHQGQGLDIYWRDNYTCPTEEEYKAMVLQKTGGLFGLAVGLMQLFSDYKEDLKPLLDTLGLFFQIRDDYANLHSKEYSENKSFCEDLTEGKFSFPTIHAIWSRPESTQVQNILRQRTENIDIKKYCVHYLENVGSFEYTRNTLKELESKAYKQIDARGGNPELVALIKHLSKMFKEENE

>*Sus scrofa* (A0A287ARC4)

MEKTQETVQRILLEPYKYLLQLPGKQVRTKLSQAFNHWLKVPEDKLQIIIEVTEMLHNASLLIDDIEDNSKLRRGFPVAHSIYGIPSVINSANYVYFLGLEKVLTLDHPDAVKLFTRQLLELHQGQGLDIYWRDNYTCPTEEEYKAMVLQKTGGLFGLAVGLMQLFSDYKEDLKPLLNTLGLFFQIRDDYANLHSKEYSENKSFCEDLTEGKFSFPTIHAIWSRPESTQVQNILRQRTENIDIKKYCVHYLENVGSFEYTRNTLKELESKAYKQIDARGGNPELVALIKHLSKMFKEENE

>*Emmonsia crescens* (A0A2B7ZC05)

MTESLTSPNPIPPRTSSTGVTNGTLDSTMSGNINGSSSRNPGYKPLPEAEWVAPLGKRRTSNPYKLSLGHTRNRSSVDGSKYKDGTWSPEKEKILLGPYDYMVQQPGKDIRRQFIAAFNRWLHVPEESIAIITKVVVMLHTASLLVDDVEDSSTLRRGVPVAHNIFGTAQTINSANYVYFLALEEVQKLRNPNVINIYVKELLNLHRGQGMDVFWRDTLTCPTEEDYLEMVGNKTGGLFRLAIKLMQAESNSGIDCVALVNLMGLIFQICDDYLNLSNSTYSKNKGPCEDLTEGKFSFPIIHSIRSQPDNLQLINILKQKTTDEEVKRYAVNYMESTGSFAYTRKTVMQLRDRALVLIDGLENLTEQRQGANQKEERSGDMVRSILNKMVDATVKNGNEEREGGG

>*Gibberella fujikuroi* (Q92236)

MIPTADPILSFNPEALPPSALHMLSLSPKAMEKMSGISNPLVSPNAIPPRTSSTGIPTSLNATPTKPVLRPVPEGDWLSQKQLSPRAQMSNAGYGVMQAPNPPPDPERYAHEDLEFTAKRSWTDEKENVVRGPYDYVISHPGKDFRAQLIGAFNVWLDVPTSSLEVITRVVGMLHESSLLIDDVQDSSELRRGFPVAHNIFGVAQTINSGNYIYFVALQELHKLNNPELITIFSDELVNLHRGQGMDLFWCDTLTCPTEEDYLEMVGNKTGGLFRLGIKLMAAEANGPSPTDCVPLVNLIGLIFQIRDDYMNLSSKEYSHNKGMCEDLTEGKFSFPVIHSIRTNPTNLQLINILKQKTSDTQIKRYAVAYMESTGSFEYTRKVLSVLIERARKMAEELDQGRGSTKGIQKILDKMAIQ

>*Neurospora crassa* (P24322)

MEHVTMAVTSSSPGPAPLSLLSNNDDFIAPFNINTKFPSAIVPPRTSSNQPISVAIPSNRISSAGLAATQQAQTRKRKASVAQISLPSMLPTSFSPYTMAPQPPQPPPNPDRFATEDFFSPSRRTWSEEKEKVLTGPYDYLNGHPGKDIRSQMVKAFDAWLDVPSESLEVITKVISMLHTASLLVDDVEDNSVLRRGFPVAHSIFGIPQTINTSNYVYFYALQELQKLKNPKAVSIFSEELLNLHRGQGMDLFWRDTLTCPTEDDYLEMVSNKTGGLFRLGIKLMQAESRSPVDCVPLVNIIGLIFQIADDYHNLWNREYTANKGMCEDLTEGKFSFPVIHSIRSNPSNMQLLNILKQKTGDEEVKRYAVAYMESTGSFEYTRKVIKVLVDRARQMTEDIDDGRGKSGGIHKILDRIMLHQEENVAQKNGKKE

>*Kwoniella mangroviensis* (A0A1B9IHN8)

MDYSNLRKTLHKPVWSDTQERTLLEPYTYISANPGKEFRGKLIDAFNIWLKVPEEDLKVVTRIVRMLHNASLLMDDVEDNSELRRGLPVAHTIYGIPQTINTANYVYFLAMQELLSLRDTTSKGKKKDVDVVGLVTDELLHLHRGQGLDLFWRDTLTCPTEKEYVDMVLGKAGGLLRLAVKLMMAKSESGVDYVPLVNLISVWFQIRDDYMNLQSTEYEANKGYCEDLTEGKFSFPVVHGVRADDSNRQILNVLQKKTTSVSLKKHVVDHLRDETKSFEYTKKIIIDLQNQIQDEIKDLGGNKYLEMAIRSLALKEDE

>*Tsuchiyaea wingfieldii* (A0A1E3HD68)

MSFQGDVDVPDYANLRQHVHNPGWSDAQERIILEPYTYISTNPGKEIRSKLIAAFNLWLDVEPGDLEVITKVVRMLHNASLLMDDVEDSSELRRGLPVAHTIYGIPQTINTANYVYFQAFQHLLELGKDRLVGRGESGSGGRGRGGEKDLVEVVNEELLQLHRGQGMDLFWRDSLTCPTEKEYVDMVLGKAGGLLRLAVKLMMAKSDSKANYVPLVNLISIWFQIRDDYMNLSSTEYESNKGYCEDLTEGKFSFPIVHGIRADPSNRQILNVLQKKTTLPSLKHHVVTYLQHTTHSFTYTRSVLKSLEGQILEEIRELGGNGMLEGVVAALSLGEEVEEGGVEEPV

>*Mucor circinelloides f. lusitanicus* (Q9P885)

MLNSHNRTEERSTEDIILEPYTYLISQPGKDIRAKLISAFDLWLHVPKDVLCVINKIIGMLHNASLMIDDVQDDSDLRRGVPVAHHIYGVPQTINTANYVIFLALQEVMKLNIPSMMQVCTEELINLHRGQGIELYWRDSLTCPTEEEYIDMVNNKTSGLLRLAVRLMQAASESDIDYTPLVNIIGIHFQVRDDYMNLQSTSYTNNKGFCEDLTEGKFSFPIIHAIRKDPSNRQLLNIISQKPTSIEVKKYALEVIRKAGSFEYVREFLRQKEAESLKEIKRLGGNPLLEKYIETIRVEATND

>*Rhizophagus irregularis* (A0A2H5T5H2)

MIIYYLTIYYLNTEMDKLEQQPWSPEKEKVLLEPYNYLLTYPGKEIRSKLIDAFDHWLKVPKEKLTIVTKVVEMLHTASLLIDDVEDDSKLRRGVPVAHSIYGVPSTINCANYVYFLGLNELTKLNDINMFNIYTEELLNLHRGQGMELYWRDTLTCPSEDEFIEMVSNKTGGLLRLGVKLMQAASESRVDYVPLVNLIGIHFQIRDDYMNLQSDKYADNKGFCEDLTEGKFSFPIIHSIHSDPDNRQLINILKQHTTSIELKQFAIKLMKESGSFDYTKHYLSQTEKKARDEVKRLGGNPMLEQLMDFLSVED

>*Saccharomyces cerevisiae* (Q12051)

MEAKIDELINNDPVWSSQNESLISKPYNHILLKPGKNFRLNLIVQINRVMNLPKDQLAIVSQIVELLHNSSLLIDDIEDNAPLRRGQTTSHLIFGVPSTINTANYMYFRAMQLVSQLTTKEPLYHNLITIFNEELINLHRGQGLDIYWRDFLPEIIPTQEMYLNMVMNKTGGLFRLTLRLMEALSPSSHHGHSLVPFINLLGIIYQIRDDYLNLKDFQMSSEKGFAEDITEGKLSFPIVHALNFTKTKGQTEQHNEILRILLLRTSDKDIKLKLIQILEFDTNSLAYTKNFINQLVNMIKNDNENKYLPDLASHSDTATNLHDELLYIIDHLSEL

>*Ashbya gossypii* (Q758K0)

MDSVEGLALGPVIWTASQEELLRQPYNHLVTQPGKNFRNTLIRVFNGFYGLSERQVAAVTELVEMLHVASLLIDDIEDNSAWRRGVAAAHVVYGSPMTINTANYMYFVSMSLLGQLAAQRPAGPLQDLLKVFNEEMMNLHRGQGLDIYWRDTFTVPSEHDYLRMVMHKTGGLFRLTVRIMEALREGPDGPGSTLVPLSNLLGVLYQVRDDYLNLTDSRMSENKGFADDITEGKFSYPIIHGLQYARVHDPAGYDFLVSVLRQRTTDITTKRRVVRYLADVSGSLAYTKQRIIELATLIKTKYIPASGTELCNVIDSLTSF

>*Oryza sativa subsp. japonica* (Q7XI92)

MAAFPPLAASRVRISPLIPAAAMAGTAGAAAASYAQHRRRFCAIVATAAASPVPAAAAAAATGFDFNAYMGEKAAAVNRALDASIPADEPPAALHEAMRYALLAGGKRVRPALCLAACAVVGGREAWAMPAAAAVEMVHTMSLVHDDLPCMDDDDLRRGKPTCHVVYGEPIAVLTGDALLSLSFHHMARFDSYPPDIDADKHPARVVRAIGELARCIGSEGLVAGQVVDLEMTGSTETVPLERLEYIHLHKTAALLEASVVIGAILGGGSDEQIESLRMYARSIGLLFQVVDDILDVTKSSEELGKTAGKDLASDKTTYPKLLGLEKSREFAEKLLSDAREQLSGFDQETAAPLLHLANYIAYRQN

>*Arabidopsis thaliana* (O04046)

MEPQILFLYLSLFILSLNFFFTNLKPRLVRLFQPSLESRVKTALLSRKEVAAFLDSPIVEDEEGEEREEEEEGGIVSNANFTFEFDPYMMSKAESVNKALEEAIPVGEPLKIHEAMRYAILAAGKRVRPILCLASCELVGGQENAAMPAACAVEMIHTMSLIKDDLPCMDNDDLRRGKPTTHKVYGEGVAILSGGALLSLAFEHMTTAEISSERMVWAVRELARSIGTRGLVAGQAMDISSEGLDLNEVGLEHLEFIHVHKTAVLLETAAVLGAIIGGGSDEEIESVRKFARCIGLLFQVVDDILDETKSSEELGKTAGKDQLAGKLTYPKLIGLEKSKEFVKRLTKDARQHLQGFSSEKVAPLVALTTFIANRNK

>*Arabidopsis thaliana* (Q9SLG2)

MEAQNIFLYLLIVFLSLHFVFTTLKGRLSPANTRRLIRLLHIPIKSPVAAAIFARKDTREFLDSSIKLVNEEDDFGFSFDFKPYMISKAETINRALDEAIPLIEPLNIHKAMRYAILAGGKRVRPILCLAACELVGGEERLAIQAACAVEMIHTMSLIKDDLPCMDNDDLRRGKPTTHKVFGESVAILSGGALLALAFEHLTEADVSSKKMVRAVKELAKSIGTKGLVAGQAKDLSSEGLEQNDVGLEDLEYIHVHKTGSLLEASAVIGAVIGGGTEKEIEKVRNFARCIGLLFQVVDDILDETKSSEELGKTAGKDKVAGKLTYPKVIGVEKSKEFVEKLKRDAREHLQGFDSDKVKPLIALTNFIANRNH

>*Gloeobacter violaceus* PCC 7421 (Q7NNJ5)

MSQFDLDAYLRQCRSQVEAALDRYLPQQYPDKLYEAMRYSLLAGGKRLRPILCLTSCRMSGGSADLAMPTACALEMVHTMSLIHDDLPAMDDDDYRRGKLTNHKVYGEDMAILAGDALLAYAFQLIAEKTVGAQPERVVEVLGRLGKAASGAGLVGGQVVDLESEGRTITLETLEYIHTHKTGALLEVSVISGAILAGADGGVVERLTRYSRCIGLAFQIRDDLLDITATQAELGKTAGKDLRDRKATYPSLLGYDGARDRAEQLREQAVAELADFGASARPLTALAEFIVQRRH

>*Phormidesmis priestleyi Ana* (A0A0P7YW11)

MVTTNLNPPSTTASTTFNLKSYLAKRATQVEAALDSSLPVVYPEQIYESMRYSLMAGGKRLRPILCLATCEMLGADSAIAMPTACALEMVHTMSLIHDDLPAMDNDDYRRGKLTNHKVYGEDVAVLAGDALLTYAFEYIATQTQGAQPQQVLKVIADLGKAVGANGLVGGQIVDLASEGKAIDEETLTYIHMHKTAALLEVSVTSAAILVGADVDVIERLRRYAQRIGLAFQIVDDVLDITATSEELGKSAGKDVAAQKATYPSLLGLEASKQKADQLIEAAIAELAGFGEAAQPLVAIAQYITARTH

>*Synechococcus* sp. PCC 7002 (B1XJV9)

MVVADAHTQGFSLAQYLQEQKTIVETALDQSLVITEPVTIYEAMRYSLLAGGKRLRPILCLAACEMLGGTAAMAMNTACALEMIHTMSLIHDDLPAMDNDDLRRGKPTNHKVYGEDIAILAGDALLSYAFEYVARTPDVPAERLLQVIVRLGQAVGAEGLVGGQVVDLESEGKTDVAVETLNFIHTHKTGALLEVCVTAGAILAGAKPEEVQLLSRYAQNIGLAFQIVDDILDITATAEELGKTAGKDLEAQKVTYPSLWGIEKSQAEAQKLVAEAIASLEPYGEKANPLKALAEYIVNRKN

>*Leptolyngbya* sp. PCC 7376 (K9Q363)

MVVDAKAQGFSLERYLKEKKAIVETALDQSIVTAEPVTVYEAMRYSLLAGGKRLRPILCLAACNMLGGTTEMAMNTACALEMIHTMSLIHDDLPSMDNDDLRRGKPTNHKVYGEDIAILAGDGLLSYAFEYVARTQGVPADRLLQVVIRLGQAVGAEGLVGGQVVDLQSEGKTDVSIETLNFIHTHKTGALLEVCVTSGAILAGAENAKIQKLQKYAQNIGLTFQIIDDILDITATQEELGKTAGKDLDAQKVTYPSLWGLEKSRSEAQRLTDEAIASLEDYGASAAPLKALAEYIINRKN

>*Synechococcus elongatus* (Q9S5F1)

MISADPPQVHPAGTFDVKAYLKERQALVEAALEASIPVAYPEKIYDAMRYSLMAGGKRLRPILCLATCELMGGTVEMAMPTACALEMIHTMSLIHDDLPAMDNDDYRRGKPTNHKVYGEDIAILAGDGLLAYAFEYVVEQTKNVPAEYLLKIVARLGHAVAATGLVGGQVVDLECEGQPDIGLETLHFIHSHKTGALLEASVVSGALLTGAHESDVARLSRYAANIGLAFQIVDDILDITSTRDVLGKTVGKDVAAQKMTYPRLWGLEKSRQEAERLVAEAKAELAVYGAAAVPLQAIADYITSRSH

>*Synechocystis* sp. PCC 6803 (P72683)

MVAQQTRTDFDLAQYLQVKKGVVEAALDSSLAIARPEKIYEAMRYSLLAGGKRLRPILCITACELCGGDEALALPTACALEMIHTMSLIHDDLPSMDNDDFRRGKPTNHKVYGEDIAILAGDGLLAYAFEYVVTHTPQADPQALLQVIARLGRTVGAAGLVGGQVLDLESEGRTDITPETLTFIHTHKTGALLEASVLTGAILAGATGEQQQRLARYAQNIGLAFQVVDDILDITATQEELGKTAGKDVKAQKATYPSLLGLEASRAQAQSLIDQAIVALEPFGPSAEPLQAIAEYIVARKY

>*Calothrix* sp. NIES3974 (A0A1Z4NGQ8)

MVAAENIQTNQTLTKFDLKGYLKERKALCEAALDASLPVQYPEKIYESMRYSLLAGGKRLRPILCLATCEMMGGTIEMAMPTACALEMIHTMSLIHDDLPAMDNDDYRRGKLTNHKVYGEDIAILAGDGLLTYAFEYPVTQTKNVPAERLLKVVAKLAKAVGAEGLVGGQVVDLECEGKTDTSLETLNYIHNHKTAALLEACVVCGGILAGASEEDIERLSRYSQNIGLAFQIIDDILDITATQEELGKSVGKDIAAQKVTYPSLWGIEKSRQQAEELIAAACQELQPYGEAAQPLIAIAHFITNRSN

>*Aliterella atlantica* CENA595 (A0A0D8ZTJ5)

MVATDNLQMNSGQAPFNLSAYLQEQQTIVEAALDRAIPVIYPEKIYEAMRYSLLAGGKRLRPILCLATCQMAGGNTETSLPTACALEMIHTMSLIHDDLPAMDNDDYRRGKLTNHKVYGDDIAILAGDGLLAYAFEFVAANTKNVPAERVLQVIARLGKAVGAAGLVGGQVVDLESEAKTDVSIETLNFIHNHKTAALLEACVVCGGILAGTSAEDLQRLSRYAQNIGLAFQIIDDILDITATQEELGKTAGKDLQAQKVTYPSLWGIEESKNQAQQLISSAISQLEPFGDKATPLIAIAHYITSRTH

>*Rivularia* sp. PCC 7116 (K9RHF4)

MVAADNLTKNETESKQSKFDLSVYLKERQKLVETALDNSIAVVYPEKIYEAMRYSLLAGGKRLRPILCLATCEMTGGNTEIAMPTACALEMIHTMSLIHDDLPAMDNDDYRRGKLTNHKVYGDDVAILAGDGLLAYAFEYVATNTKNVPPTQILQVISRLGRAVGAAGLVGGQVVDLEMEGKTDVSLETLNFIHNHKTAALLEASVVCGGILSGTSPEDIQKLQRYSQNIGLAFQIVDDILDITATQEQLGKTAGKDLEAQKVTYPSLWGLEESKKQAQLLVEAACAELEPFGEQAKPLVALAYFITSRSN

>*Nostoc* sp. PCC 7120 (Q8Z085)

MVAADNLRKMSEEAKFNLVAYLKERQKLCEAALDAAIPIIYPEKIYESMRYSLLAGGKRLRPILCLATSEMMGGTIEIAMPTACAVEMIHTMSLIHDDLPAMDNDDYRRGMLTNHKVYGEDIAILAGDGLLAYAFEFVAIRTPESVPRDRVLQVVARLGRALGAAGLVGGQVVDLDSEGKSDTSLETLNFIHNHKTAALLEACVVCGGILAGASSENVQRLSRYSQNIGLAFQIIDDILDITSTQEQLGKTAGKDLLAKKVTYPSLWGIEQSRVKAQQLIEAACTELEPFGEDAQPLKAIAHFITSRNH

>*Anabaena* sp. 90 (K7WTZ6)

MVATDKRLETPKKSESASFNLATYLKQRQQLCEQALDNSITVVYPEKIYEAMRYSLLAGGKRVRPILCLATADMIGGTIEMAMATACAVEMIHTMSLIHDDLPAMDNDDYRRGKLTNHKVYGEDVAILAGDGLLALAFEHVAAQTPTNVPRDRVLQVIIRLGKALGAAGLVGGQVVDLESEGKSDISLETLNFIHNHKTAALLEACVVCGGIIAGASAEDVQKLTRYSQNIGLAFQIIDDILDITATQEQLGKTAGKDLTAQKVTYPSLWGIEESRAKAQHLVAQACAELASYGEKAIPLQALAHFIINRNN

>*Coleofasciculus chthonoplastes* PCC 7420 (B4VH16)

MVATDGRPAPNGESPAFDLSTYLKERKAQVEAALDRSLPLGYPEKIYEAMRYSLLAGGKRLRPILCLATCELMGGTMEMAMPTACALEMLHTMSLIHDDLPAMDNDDYRRGKLTNHKVYGEDMAILAGDGLLAYAFEYVATQTQGVPAERVLQVIARLGRTVGAAGLVGGQVVDLESEGKSDIQEETLHYIHTHKTGALLETCVVCGGILAGATDADIERLSRYAQNIGLAFQIIDDILDITATQEELGKTAGKDLQAQKATYPSLWGLEASKAKAQELIEEAIAQLEPFGERSQPLIALANFITARTY

>*Gloeocapsa* sp. PCC 73106 (L8LKD0)

MVKANERLSDKNIDSRFDLKTYLDAQKTIIEAALDRSLSISKPEKIYEAMRYSLLAGGKRLRPILCLATCELMGGTSVIALPTACALEMIHTMSLIHDDLPAMDNDDYRRGKLTNHKVYGDDIAILAGDGLLAYAFEYVATQTQGVPPERLIKVIIHLSHAVGAEGLVGGQVLDLESEGKTDISAETLTFIHTHKTGALLEACVLSGADLAGANREDLERLSKYAQNIGLAFQIIDDILDITATVEELGKTAGKDLKAQKVTYPSLWGIEASQTQAQQLVEGAIAQLTVYGEKADPLRAIASYIVTRKH

>*Crocosphaera watsonii* WH 8501 (Q4BXI3)

MVMTGEDRVTHTEASNFELASYLKEKKQLVEAALDQSLHITRPEKIYEAMRYSLLAGGKRLRPILCLTTCELMGGTVEMSLPTACALEMIHTMSLIHDDLPAMDNDDYRRGKLTNHKVYGEDIAILAGDALLSYAFEYVATQTCNVDFPNLIDVIARLGRTVGAAGLVGGQVLDLESEGRTDVTVDTLKFIHIHKTGALLETSVVSGAILAGAKPEDIERLSRYAQNIGLAFQIIDDVLDVTASSEELGKTAGKDERAQKATYPSLWGLEESQKQAQALIDDAIAQLSPYGQAAQPLEAIAQFIVARKN

>*Cyanothece* sp. ATCC 51142 (B1WS34)

MVMTGEDRVTQTETSDFDLASYLKEKKQLVEAALDQSLPITRPEKIYEAMRYSLLAGGKRLRPILCLTTCELMGGTREMSLPTACALEMIHTMSLIHDDLPAMDNDDYRRGKLTNHKVYGEDIAILAGDALLSYAFEYVATQTRNVDLANLMDVIARLGRTVGAAGLVGGQVLDLESEGRADVTVDTLKFIHIHKTGALLETSVVSGAILAGAKPEDIERLSRYAQNIGLAFQIIDDILDVTASTEELGKTAGKDQRDQKATYPSLWGLEESQKQAQALIDDAIAQLSPYGQSAQPLTAIAQFIVARKN

>*Stanieria cyanosphaera* PCC 7437 (K9XXZ9)

MVQTDEKLVEQEQTPVFDEVEADSRSTFDLNSYLKQQKILVEQALDSSLPINQPAKIYEAMRYSLLAGGKRLRPILCLATCELTGGSNEMAIPTACALEMIHTMSLIHDDLPAMDNDDYRRGKLTNHKVYGEDIAILAGDGLLAYAFEYVATQTKNVPAERIVKVIARLGRTVGAEGLVGGQVLDLESEGKSDISAETLTFIHTHKTGALLEASVVSGAILSGASEEDLARLSKYAQNIGLAFQIIDDILDITATDEQLGKTAGKDLQAQKATYPSLWGIEKSQHQAEKLINEAIAQLKPYGNKAKPLEAIARFIVTRNN

>*Xenococcus* sp. PCC 7305 (L8LWR1)

MVKTDQKATAQLEKPTTFDLNSYLKEQKALVETALDQSLAIGIPEKIYESMRYSLLAGGKRLRPILCLATCRLTGGSTEMAMPTACALEMIHTMSLIHDDLPAMDNDDYRRGKLTNHKVYGEDVAILAGDGLLAYAFEYVANKTDNVAPENIIKVIARLGNTVGAEGLVGGQVLDLESEGKTDITAETLSFIHTHKTGALLETSVVTGAILAGASAEEIIKLTKYSQNIGLAFQIIDDILDITATDEQLGKTAGKDLAAQKATYPSLWGLEKSQAEAEKLIAEALDQLASYGEAAEPLRAIAKFIVTRNH

>*Microcystis aeruginosa* NIES843 (B0JU89)

MVTRGEQGVEVRKNESFSLASYLEEKRLIVESALDSSLVIGNPAKIYEAMRYSLLAGGKRLRPILCLATCELMGGSLEMALPTACALEMIHTMSLIHDDLPAMDNDDYRRGKLTNHKVFGEDIAILAGDGLLAYAFEYVATQTRNVPPLQILEVIARLGRTVGAAGLVGGQVLDLESAGKPDISAETLGFIHTQKTGALLETSVVSGAVLAGAKAEDIAKLARYAQNIGLAFQIIDDVLDITATKEELGKTAGKDLQAQKATYPSLWGIEESRQQAQQLIDSAIGELSGYDEAAEPLRAIAEYIVNRKN

>*Pleurocapsa* sp. PCC 7327 (K9T847)

MVTTDEIVAEEKQSSNFDLASYLEEKKQLVEAALERSLSIGKPEKIYEAMRYSLLAGGKRLRPILCLATCELTGGTTEMAMPTACALEMIHTMSLIHDDLPAMDNDDYRRGKLTNHKVFGEDIAILAGDGLLAYAFEYVAAQTQNVPPQRILDVIARLGRTVGAAGLVGGQVLDLESEGKSDISVDTLTFIHTHKTGALLETSVVSGAMLAGASDDEIQRLSKYAQDIGLAFQIVDDILDITATQEELGKTAGKDLQAQKATYPSLWGLEESRKQAQQLVDAAIAELASYGEGAEPLRAIASYIVTRKH

>*Salvia splendens* (A0A4D8ZW25)

MALVAAIPIYEPFFPKAHPYLHHSNTRIISCKKITPTNATKTTSSSPSPQQTLASPNFKFEEYMKTKAKKVNKALDDAVPLQNPVKIHEAMRYSLLAGGKRVRPVLCLASCELVGGDEAAAVPMACALEMIHTMSLIHDDLPCMDDDDLRRGRPTSHKAFGEETAVLAGDALLSLAFEHVAAKTTNVGHERVLRAIAELGSAVGSEGLVAGQIVDLTSEGKEVSLDVLEYIHVHKTSKLLEAAVVCGAIVGGGDGEEVEALRRYARCVGLLFQVVDDILDVTKSSAELGKTAGKDLATDKATYPRLMGLGEAKEFAGELAEKAVQELAFFDTLRAAPLHHLAQYIASRQN

>*Salvia miltiorrhiza* (D7F2D7)

MRSMNLVDAWVQNLSIFKQPCPSKSLVGFIHHPRFEPVFLKSRKRISSHGVSAVLTGEEARVSTQRDDAPFNFNAYVVEKANHVNKALDDAVAVRNPPMIHDAMRYSLLAGGKRVRPMLCIAACEIVGGPQSAAIPAACAVEMIHTMSLIHDDLPCMDNDDLRRGKPTNHKVFGEDVAVLAGDALLAFAFEFMATATTGVAPERILAAVGELAKAIGTEGLVAGQVVDLNCTGDANVGLDTLEFIHIHKTAALLEASVVLGAILGGGSSDQIEKLRTFARKIGLLFQVVDDILDVTKSSEELGKTAGKDLAVDKTTYPKLLGLDKAMEFAEKLNEEAKAQLAGFDPSKAAPLTALADYIAHRQN

>*Capsicum annuum* (P80042)

MRSMNLVDLWAQQACLVFNQTLSYKSFNGFMKIPLKNSKINPKLNKKRPFSPLTVSAIATTKEDERIEAAQTEEPFNFKIYVTEKAISVNKALDEAIIVKEPHVIHEAMRYSLLAGGKRVRPMLCLAACELVGGNQENAMAAACAVEMIHTMSLIHDDLPCMDNDDLRRGKPTNHKIYGEDVAVLAGDSLLAFAFEHIVNSTAGVTPSRIVGAVAELAKSIGTEGLVAGQVADIKCTGNASVSLETLEFIHVHKTAALLESSVVLGAILGGGTNVEVEKLRRFARCIGLLFQVVDDILDVTKSSEELGKTAGKDLVVDKTTYPKLLGLEKAKEFAAELNREAKQQLEGFDSRKAAPLIALADYIAYRDN

>*Abies grandis* (Q8LKJ1)

MAYSGMVRSFQGVYFMAVALDRNRNLKRIDIPSKRFDGVSTSFVACNGEHLGLPVNLKKEFLSCIQRASSSRSSNTIVQFANLPEQGKKVVEFDFNKYMLSKAMAVTEALDKAIPHSYPQKIHESMRYSLLAGGKRVRPVLCIAACELVGGREELAMPTACAMEMIHTMSLIHDDLPCMDNDDLRRGKPTNHKVFGQDTALLAGDALHAFAFEHIVAFTSKSVGSDGILRAVTELARATGPQGIMGGQIVDIASERDAFVDLQTLEWIHIHKTAVLFECATVCGAIIGGASGDEIERIRRFARYLGLLFQVVDDILDVTKSSEDLGKTAGKDLVSDKATYPKLMGLEKAKGFSLELLNKAKEELSCFDPMKAAPLFGLADYMALRQN

>*Picea abies* (B1A9K9)

MAYSGMAASYHGLHFMTIATQECSLKRGSIPSRRFHGVPTSLWSSNGFQGHLKRQLSAYTFLVSSSRCSSTIAQLANLREEVKEVIKFDFKEYLLSKAMAVNEALDRAVQLRYPEKIHEAMRYSLLAGGKRVRPVLCIAACELVGGTEKLAMPTACAMEMIHTMSLIHDDLPCMDNDDFRRGKPTNHKVFGEGTAVLAGDALLSFAFEHIAVSTSKSVGSDRILRVVSELGRTIGSQGLVGGQVADITSEGNASVDLDTLEWIHIHKTAVLLECSVVCGAIISGASENEIERVKRYARSVGLLFQVVDDILDVTKSSKELGKTAGKDLISDKATYPKLMGLEKAKQFAVELLDRAKENLSCFDPMKAAPLLGLADYIAFRQN

>*Arabidopsis thaliana* (P34802)

MASVTLGSWIVVHHHNHHHPSSILTKSRSRSCPITLTKPISFRSKRTVSSSSSIVSSSVVTKEDNLRQSEPSSFDFMSYIITKAELVNKALDSAVPLREPLKIHEAMRYSLLAGGKRVRPVLCIAACELVGGEESTAMPAACAVEMIHTMSLIHDDLPCMDNDDLRRGKPTNHKVFGEDVAVLAGDALLSFAFEHLASATSSDVVSPVRVVRAVGELAKAIGTEGLVAGQVVDISSEGLDLNDVGLEHLEFIHLHKTAALLEASAVLGAIVGGGSDDEIERLRKFARCIGLLFQVVDDILDVTKSSKELGKTAGKDLIADKLTYPKIMGLEKSREFAEKLNREARDQLLGFDSDKVAPLLALANYIAYRQN

>*Arabidopsis thaliana* (Q9ZU77)

MTTLNLSIFPSVKISSSASIPGFIKIQPFLLRRKLSTVLSVTARDEGIIHNHFDFTSYMIGKANAVNEALDSAVSLREPIKIHEAIRYSLLARGKRVRPVLCIAACELVGGEESVALPAACAVEMIHTMSLIHDDLPCMDNDDLRRGKPTNHKVFGEDVAVLAGDALISFAFEHLATSTAVSPARVVRAIGELAKAIGSKGLVAGQVVDLTSGGMDQNDVGLEVLEFIHVHKTAVLLEAATVLGAIVGGGSDEEVEKLRRFARCIGLLFQVVDDILDVTKSSEELGKTAGKDLIADKLTYPKLMGLEKSKDFADKLLSDAHEQLHGFDSSRVKPLLALANYIAKRQN

>*Mentha piperita* (Q9SBR3)

MSALVNPVAKWPQTIGVKDVHGGRRRRSRSTLFQSHPLRTEMPFSLYFSSPLKAPATFSVSAVYTKEGSEIRDKDPAPSTSPAFDFDGYMLRKAKSVNKALEAAVQMKEPLKIHESMRYSLLAGGKRVRPMLCIAACELVGGDESTAMPAACAVEMIHTMSLMHDDLPCMDNDDLRRGKPTNHMAFGESVAVLAGDALLSFAFEHVAAATKGAPPERIVRVLGELAVSIGSEGLVAGQVVDVCSEGMAEVGLDHLEFIHHHKTAALLQGSVVLGAILGGGKEEEVAKLRKFANCIGLLFQVVDDILDVTKSSKELGKTAGKDLVADKTTYPKLIGVEKSKEFADRLNREAQEQLLHFHPHRAAPLIALANYIAYRDN

>*Antirrhinum majus* (Q6QLV1)

MSLVNPITTWSTTTTSKSPKNVQTTTRSRSIILPHKISLFPSNPKSKSKTHLRFSISSILTKNPQESSQKTSKDPTFTLDFKTYMLEKASSVNKALEQAVLLKEPLKIHESMRYSLLAGGKRVRPMLCIAACELVGGLESTAMPSACAVEMIHTMSLIHDDLPCMDNDDLRRGKPTNHKIYGEDVAVLAGDALLAFSFEHVAKSTKGVSSDRIVRVIGELAKCIGSEGLVAGQVVDISSEGMTEVGLEHLEFIHVHKTAALLEASVVLGAIVGGADDEDVEKLRKFARCIGLLFQVVDDILDVTKSSQELGKTAGKDLVADKTTYPKLLGIEKSREFAEKLNREAQEQLEGFDSVKAAPLIALANYIAYRDN

>*Ostreococcus tauri* (Q00Y25)

MRSTARAPSARVVARRAIGGRRGTDARRARTEASAPATSGASSTSADSEREFNAYIKGVNAKIERALDASVPVHYPERLSESMRYSLLAGGKRIRPALCLAACELVGGDMETAMPTACALEMIHTMSLIHDDLPSMDNDDFRRGKPTNHKVYGEEMAILAGDALLSHAFEYIGRETKGASAEKVLRVIVDVGKCVGSEGLVGGQVVDIMSEGAGPENVTVDTLKYIHAHKTGALLEVSVTAGAILGGASEEQIGKLERYAQNIGLAFQVVDDILDCTASSEELGKTAGKDVAVGKTTYPSLVGMEESKRIAADLISKAKAELEGFDPVKVRPLIGLADYIQNRKN

>*Micromonas pusilla* CCMP1545 (C1MHH2)

MQAILCAPGAIIARLNPRQKKVNNLRQKTQHLRAAAAPAAAVEASELDFGNYIADKNKAIEQALSDSVPEMYPERLHESMRYSLLAGGKRIRPCLCIAACELMGGTMEVAMPTACALEMIHTMSLIHDDLPSMDNDDFRRGKPTNHKVYGEEMAILAGDALLSHAFEYIGRETPKSVPAERTLRVLVDVGKCVGSEGLVGGQVVDIMSEGADADEVTLETLKYIHAHKTGALLEVSVTSGAILAGAKEEDVERLGKYAQLIGLAFQVVDDILDCTQTSEQLGKTAGKDEAVGKATYPSLVGLEESKKIADDLITEAKELLRDYDPKLAAPLLGLADFIKNRQN

>*Micromonas commoda* RCC299 (C1EAR1)

MQAVCLSTPAAIVRSSVRGRARSKSVVAPRRGGVPSVRAAAEAPAAAVEASELDFAAYIAEKNAAIEQALSDSVPEMYPERLHESMRYSLLAGGKRIRPCLCIAACELMGGTMDVAMPTACALEMIHTMSLIHDDLPSMDNDDFRRGKPTNHKVYGEEMAILAGDALLSHAFEYIGRETPKSVPAERTLRVIVDVGKCVGSEGLVGGQVVDIMSEGAEAGEVTVETLQYIHAHKTGALLEVSVTAGAILAGAKEEDVERLGKYAQLIGLAFQVVDDILDCTQTSEQLGKTAGKDEAVGKATYPSLVGLEESKKIADKLIADAKDLLKDYDQEKAAPLIGLADYIKNRQN

>*Tetraselmis* sp. GSL018 (A0A061RXB5)

MSVVSKSMVPRANCACSAALPARPKVTVSSFAKPVLSPYGIKLVDTASQRTLRRAAAAEPATSSVDDGFTFDFKQYMLKRAQEMEVALDQSIQPHYPEVISESMRYALLGGGKRIRPALCLAACELVGGDPGVAMPTACALEMIHTMSLIHDDLPAMDNDDFRRGKPTVHKVYGDDMAILSGDAMLVRAFEYVARETKGAPAERVLRVIADLGKAVGSEGLVGGQAVDIQMEGANEEASLETLNYIHTHKTGALLEAAVVSGAVLGGATEPEIEKLRKYALDIGLAFQVIDDILDVTKTTEELGKTAAKDLASDKTTFPKLFGLERSREIAEELISSAKAQLAGYPVEKAAPLLGLAD

>*Raphidocelis subcapitata* (A0A2V0NYH2)

MQTQLRSLRQRACGGARCTARSSAPAAAARRSSRAQVRIHVAAPTTEAVSSSEPGTFDFKSYMESTAASVNAALDAACPSKYPETLNEAMRYSLLAGGKRVRPALCLAACEMVGGDVAAAMPTACAMEMVHTMSLIHDDLPAMDNDDFRRGRPTNHKVYGENMAILAGDAMLTFAFEHVARATKGVPADRVLRVIAELGRASGADGLVGGQVVDIQSEGKEVGLDVLRYIHEHKTAALLEAAVVCGAIVGGASDEDVEKLRRYALNIGLAFQVVDDILDITQSTEVLGKTAAKDLAVNKTTYPKLLGLEKSREVAEQLISEAIAQLDGFEPAKAAPLVALAKFIGYRQN

>*Chlamydomonas reinhardtii* (A8JHU6)

MQMQQQRMRFSRDARRIGASRVPLAKAAPGRKVVAQVATATAEKVDVKQAGSFDFNTYMVDRAKLVNKAMDEAVPLKYPETLNESMRYSLLAGGKRVRPALCLAACELVGGDIHAALPAACAMEMVHTMSLIHDDLPSMDNDDFRRGRPTNHKVYGEDIAILAGDALLSFSFEHIARATKGVPAERVLRVIMELGKAVGQDGLVAGQVVDIQSEDKEVGLDVLKYIHEHKTAALLEASVVCGAILGGADESTIEKLRKYSLNIGLAFQVIDDILDVTATTEQLGKTAAKDLAVNKTTYPKLLGLEKSKQVADDLIKEAIQQLDGFDAAKAAPLVALAKFIGYRQN

>*Chlamydomonas eustigma* (A0A250XAK8)

MLQFGKSSIRELRGTKQPIHRVPMQCMRRRLLHVATAEAVTTRPIEAAQFDFESYMVERAKLVNKALDQAVPLVYPETLTEAMRYSLLAGGKRVRPALCLAACDLVGGNLESAMPAAIAMEMVHTMSLIHDDLPAMDNDDFRRGRPTNHKVYGEDVAILAGDALLTFSFEHIAKSTKEVPAERVLRVIVELGRAVGAEGLTAGQVVDIKSENQEVGLDVLQYIHHHKTAALLEAAVVCGAIVGGADEATVEKLRKYALNIGLAFQVVDDILDVTQTTEQLGKTAAKDLAVNKTTYPKLLGIEKSREIADDLIKEAIQQLDGFESSRAAPLIALAKFIGYRQN

>*Dunaliella viridis* (V9HXF1)

MWALKRGATCCPSQRPTPSCSRPAAAWRVSRSSCRTLAIATADGAAKQSTASFDFKGYMKQRAVMVNEALDKALPRRHPEVLLDSMRYSLLAGGKRVRPALTLAACEMVGGSIETAMPTACAMEVVHTMSLIHDDLPSMDNDDFRRGNPTNHKVYGEDMAILAGDALLSFAFEHVARATKGTSPERVLRVILELGRAVGADGLTGGQVVDIKSENEEVGLEVLQFIHEHKTAALLEASVVCGALVGGADDVTVEKLRKYARNIGLAFQVVDDILDCTQTTEMLGKTAGKDLDVNKTTYPKLLGLEKSKQTAEDLISEAIQQLDGFAPEKRVPLVALAKYIGYREN

>*Dunaliella salina* (A0A1P8LG29)

MAAHQMQLLNSQRLCSTSTRSIRPAVSNRPQVPRRPANVRRGRYQACRTMAIATADEAKQSTSSFDFQGYMMERAVMVNDALDKALPQRHPEVLLDAMRYSLLAGGKRVRPALTLAACELVGGDIACAMPTACAMEVVHTMSLIHDDLPSMDNDDFRRGRPTNHKVYGEDIAILAGDALLSFAFEHVARATTGTSPERVLRVILELGKAVGADGLTGGQVVDIKSENEEVGLEVLQYIHEHKTAALLEASVVCGALVGGADDVTVEKLRKYARNIGLAFQVVDDILDCTQTTEMLGKTAGKDIDVNKTTYPKLLGLEKSKQAAEDLIAEAIQQLDGFPPEKRTPLVALAKYIGYRQN

>*Coccomyxa subellipsoidea* C169 (I0Z6E1)

MKERATLVNEALDKSVPLQYPEIINESMRYSLLAGGKRVRPCLCLAACEAVGGNIAQAMPTACAMEMLHTMSLIHDDLPSMDNDDFRRGKPTNHKVYGEDVAILAGDALLCLAFEYICRETRGVAPERIVRVVCEVGKATGAEGLVAGQIVDIKSEGMAEQVGLETLQYIHEHKTAALLEASVVCGALVGGAGEPDVERLRKYARCIGLAFQVVDDILDITQSSEVLGKTAGKDLLSDKTTYPSLLGIERSKEVAQQLIADAKEQLSCYDPAKVAPLLGLATYIGARTN

>*Micractinium conductrix* (A0A2P6VD43)

MKDRAVLIDAALDKSVPLQYPEVINEAMRYSLLAGGKRVRPALCLAACELVGGTIEQAMPTACSLEMIHTMSLIHDDLPSMDNDDFRRGKPTCHKVYGEEIAILAGDALLSLSFEYIARETRGVDAQRVLQVVVEVGKAVGSEGLVAGQVVDIKSEGAGATVGIDTLQYIHEHKTAALLEAAVVSGAILGGANEMDVDRLRKYSRSIGLAFQVVDDILDITATTEELGKTAGKDLASAKTTYPALVGLERSREIADELIEEAKSMLTGYDKMKAAPLFALAEYIRSRKN

>*Auxenochlorella protothecoides* (A0A087SJF4)

MKTFALTVLLLGTAYGAAAVKPGEWWVYPFLSTAVDHQKRVLQVEGDGRLGADMFCKSKGYLKSGDFLELEEAGNIWPIGTTNLANDQFWTGANATAYLYITCLSLETQSPSIGQGENGNIGYKNTGINNIGNYNSGEANVGNHNAGDFNMGDGQHSARYVPRRAARSRGHPTRCTAEVRARPSANGAQPMTAFDFRQYMQQRAALVDAALDLAVPLQYPEKINEAMRYSLLAGGKRVRPALCLAACELVGGPLEAAMPAACAMEMIHTMSLIHDDLPAMDNDDFRRGQPANHKAYGEEIAILAGDALLSLSFEHIARETRGVDPVRVLAAISEVGRAVGSRGLVAGQVVDLGSEGGGVGLATLRYIHEHKTAALLEAAVVSGALLGGAEEADLERLRTYSRAIGLAFQVVDDILDITGTSEELGKTAGKDLSSAKTTYPSLVGLARSREIADELIEDAKAQLTQYEPARAAPLVALAEYIRNRKN

>*Sulfolobus acidocaldarius* ATCC 33909 (P394640)

MSYFDNYFNEIVNSVNDIIKSYISGDVPKLYEASYHLFTSGGKRLRPLILTISSDLFGGQRERAYYAGAAIEVLHTFTLVHDDIMDQDNIRRGLPTVHVKYGLPLAILAGDLLHAKAFQLLTQALRGLPSETIIKAFDIFTRSIIIISEGQAVDMEFEDRIDIKEQEYLDMISRKTAALFSASSSIGALIAGANDNDVRLMSDFGTNLGIAFQIVDDILGLTADEKELGKPVFSDIREGKKTILVIKTLELCKEDEKKIVLKALGNKSASKEELMSSADIIKKYSLDYAYNLAEKYYKNAIDSLNQVSSKSDIPGKALKYLAEFTIRRRK

>*Geoglobus acetivorans* (A0A0A7GEY4)

MISEIIKDRAKLVNEKIEELLKEQEPEGLYRAARHYLKAGGKRLRPVITLLSAEALGEDYRKAIHAAIAIETVHNFTLVHDDIMDEDEMRRGVKTVHTLFGIPTAILAGDTLYAEAFEILSMSDAPPENIVRAVSKLARVCVEICEGQFMDMSFEERDSVGESEYLEMVRKKTGVLIGISASIPAVLFGKDESVEKALWNYGIYSGIGFQIHDDLLDISGKGKIGKDWGSDILEGKKTLIVIKAFEEGIELETFGKGRASEEELERDIKKLFDCGAVDYARERAREYIEMAKKNLEVIDESPSRNYLVELADYLIERDH

>*Natronomonas pharaonis* ATCC 35678 (Q3IPL1)

MTSADHVESAIAERREIVNEAVSEQLPVQKPERLYSASRYLLDAGGKRLRPTILLLAAESLADVEPLSADYRQFPSLPGDEVDVLSAAVSIEVIQSFTLIHDDIMDDDDLRRGVPAVHREYDLETAILAGDTLYSKAFEYMLDTGAPAERSVEALDELATTCTEICEGQALDVDFENRSDVTTEEYLEMVEFKTAVLYAAAASIPAILLGSDDETVEALHGYGLDIGRAFQIQDDLLDLTAPSDELGKQRGSDLVENKRTVITLHARDQGIDVEGLVSDDPSDAEIEAAVQTLEDAGSIDFAREMALDLVTSGKERLDVLPENEARQLLEDIADFLVERSY

>*Methanosarcina mazei* (Q8PW340)

MMLMTLVDEIKNRSSHVDAAIDELLPVTRPEELYKASRYLVDAGGKRLRPAVLILAAEAVGSNLRSVLPAAVAVELVHNFTLIHDDIMDRDDIRRGMPAVHVKWGEAGAILAGDTLYSKAFEILSKVENEPVRVLKCMDVLSKTCTEICEGQWLDMDFETRKKVTESEYLEMVEKKTSVLYAAAAKIGALLGGASDEVAEALSEYGRLIGIGFQMYDDVLDMTAPEEVLGKVRGSDLMEGKYTLIVINAFEKGVKLDIFGKGEATLEETEAAVRTLTECGSLDYVKNLAISYIEEGKEKLDVLRDCPEKTLLLQIADYMISREY

>*Methanothermobacter thermautotrophicus* (O26156)

MMEVMDILRKYSEMADERIRESISDITPETLLRASEHLITAGGKKIRPSLALLSSEAVGGDPGDAAGVAAAIELIHTFSLIHDDIMDDDEIRRGEPAVHVLWGEPMAILAGDVLFSKAFEAVIRNGDSEMVKEALAVVVDSCVKICEGQALDMGFEERLDVTEEEYMEMIYKKTAALIAAATKAGAIMGGGSPQEIAALEDYGRCIGLAFQIHDDYLDVVSDEESLGKPVGSDIAEGKMTLMVVKALERASEKDRERLISILGSGDEKLVAEAIEIFERYGATEYAHAVALDHVRMAKERLEVLEESDAREALAMIADFVLEREH

>*Pyrococcus horikoshii* ATCC 700860 (O58799)

MEKYEELFARIKEKAKLIDEKIFELIPEKDPRVLYEAARHYPLAGGKRVRPFVVLTSTEAVGGDPLRAIYPAVAIELIHNYSLVHDDIMDMDETRRGKPTVHRIWGVNMAILAGDLLFSKAFEAVARAEIPPEKKARVLEVIVKASNELCEGQARDLEFEKKSTVTIEEYMEMISGKTGALFEASAKVGGIIGTDNEEYIKALSSWGRNVGIAFQIWDDVLDLIADEKKLGKPVGSDIRKGKKTLIVAHFFENADEKDKQRFLKIFGKYAGDVKGRGIIEEDIKSDVMEAIDLLKKYGSIDYAAEIAKDMIKKANEALRILPKSKARMDLELLAKFIVEREY
